# Circuit-Level Modeling and Simulation of Wireless Sensing and Energy Harvesting With Hybrid Magnetoelectric Antennas for Implantable Neural Devices

**DOI:** 10.1109/ojcas.2023.3259233

**Published:** 2023-03-20

**Authors:** DIPTASHREE DAS, ZIYUE XU, MEHDI NASROLLAHPOUR, ISABEL MARTOS-REPATH, MOHSEN ZAEIMBASHI, ADAM KHALIFA, ANKIT MITTAL, SYDNEY S. CASH, NIAN X. SUN, AATMESH SHRIVASTAVA, MARVIN ONABAJO

**Affiliations:** 1 Department of Electrical and Computer Engineering, Northeastern University, Boston, MA 02115, USA; 2 The Wellman Center for Photomedicine, Massachusetts General Hospital, Harvard Medical School, Boston, MA 02115, USA; 3 Department of Electrical and Computer Engineering, University of Florida, Gainesville, FL 32611, USA; 4 Department of Neurology, Massachusetts General Hospital, Harvard Medical School, Boston, MA 02115, USA; 5 MediaTek Inc., Woburn, MA 01801, USA

**Keywords:** Behavioral circuit modeling, magnetoelectric antenna, wireless energy harvesting circuits, wireless sensing, neural recording

## Abstract

A magnetoelectric antenna (ME) can exhibit the dual capabilities of wireless energy harvesting and sensing at different frequencies. In this article, a behavioral circuit model for hybrid ME antennas is described to emulate the radio frequency (RF) energy harvesting and sensing operations during circuit simulations. The ME antenna of this work is interfaced with a CMOS energy harvester chip towards the goal of developing a wireless communication link for fully integrated implantable devices. One role of the integrated system is to receive pulse-modulated power from a nearby transmitter, and another role is to sense and transmit low-magnitude neural signals. The measurements reported in this paper are the first results that demonstrate simultaneous low-frequency wireless magnetic sensing and high-frequency wireless energy harvesting at two different frequencies with one dual-mode ME antenna. The proposed behavioral ME antenna model can be utilized during design optimizations of energy harvesting circuits. Measurements were performed to validate the wireless power transfer link with an ME antenna having a 2.57 GHz resonance frequency connected to an energy harvester chip designed in 65nm CMOS technology. Furthermore, this dual-mode ME antenna enables concurrent sensing using a carrier signal with a frequency that matches the second 63.63 MHz resonance mode. A wireless test platform has been developed for evaluation of ME antennas as a tool for neural implant design, and this prototype system was utilized to provide first experimental results with the transmission of magnetically modulated action potential waveforms.

## I. INTRODUCTION

**FIGURE 1.**
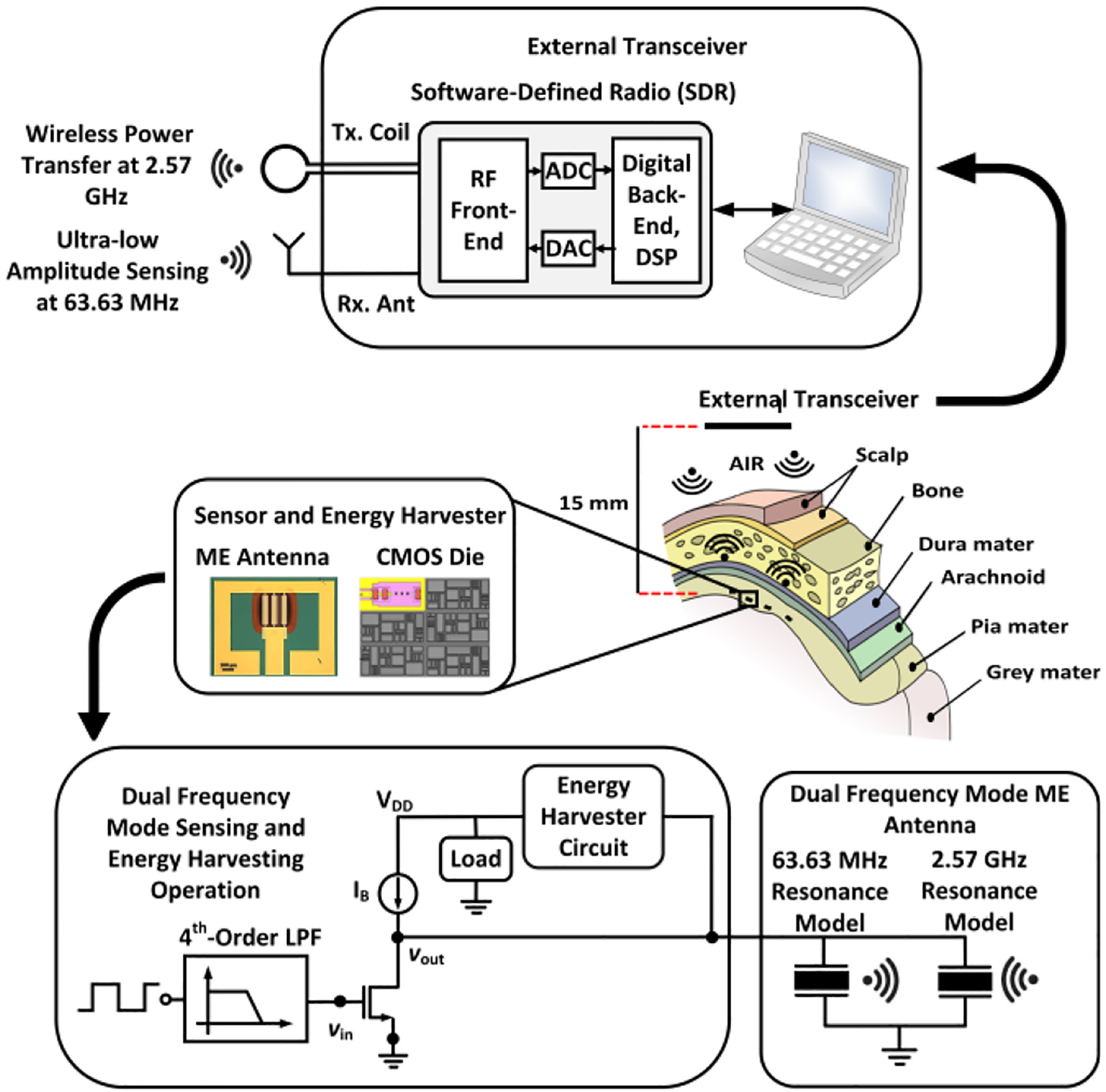
Conceptual representation of the envisioned wireless energy harvesting and sensing application with a hybrid ME antenna and a systemon-a-chip (SoC) containing circuits for energy harvester and sensing.

WIRELESS Sensing has emerged as a viable option for implantable medical devices (IMDs) [1] with an essential need for power and device size minimizations. Numerous works and approaches for neural recording and wireless transmission/reception using integrated CMOS circuits have been reported with robust functionalities [2], [3], [4], [5], [6], [7], [8], [9], [10], [11], [12], [13]. Power-efficient brain machine interface circuits for neural recording with feature extraction have also been reported [14], [15]. In parallel to sensing and processing neural signals, highly efficient wireless power transfer (WPT) for energy harvesting is equally crucial for practical IMDs [16], [17], [18], [19], [20], [21], [22], [23], [24], [25]. Energy harvesting techniques are also extensively employed within wakeup receivers (WRXs), which are a popular choice to reduce the average power consumption. WRXs are used to enable the active mode of devices through an RF-based wakeup request when needed [26], [27], [28]. For example, a WRX with improved sensitivity has been reported in [29], which incorporated an energy harvester-based power management unit to support three different wakeup modes. The continuous miniaturization of device sizes will benefit the expansion of WRX-based wireless body area networks with energy harvesting [30]. Implementation of miniaturized wireless neural monitoring devices will account for understanding of complex brain functions that requires sensing of ultra-low frequency and ultra-low amplitude neural information. Descriptions of complex neural functions and the challenges to implement circuit-level configurations are provided in [31]. Based on the associated brain locations, neural signals can be classified into multiple categories: electroencephalogram (EEG) [32], electrocorticogram (ECoG) [33], and action potentials (AP) [34]. With primary goal of advancing deep brain monitoring and stimulation applications as visualized in Fig. 1, action potentials have been selected in this work as reference for neural modulation during wireless sensing based on [31], [35]. Fig. 1 shows the measurement setup which includes a miniaturized hybrid magnetoelectric (ME) antenna-sensor, CMOS integrated circuits and a software defined radio (SDR) for executing neural modulation and accurate signal reconstruction wirelessly. To enhance measurement and modeling techniques, customized test setups are gaining increasing interest among circuit designers involved in interdisciplinary research. For example, [36] includes a measurement test setup to characterize Lorentz-force MEMS magnetometers. On the other hand, [37] describes the difficulties of collecting data involving measurements with flexible printed circuit boards (PCBs) for wearable healthcare monitoring applications. As part of this research, a measurement platform was developed to experimentally investigate and demonstrate phenomena that enable magnetic modulation by exploiting the properties of micrometer-sized ME antennas. The platform allows wireless sensing and reconstruction of signals together with wireless power transfer, which can be utilized during the co-design and optimization of ME antennas and integrated circuits for various applications.

For IMD applications, the need of wireless power transfer and wireless transmission of neural signals calls for small-sized antennas and sensors interfaced to systems-on-a-chip (SoCs). High-level integrations of CMOS technology with electromagnetic ([38], [39], [40]) and magnetic technologies ([41], [42]) including magnetothermal, magnetoelectric ([43], [44], [45]) and inductive coupling [46] showed great potential for the development of minuscule and contactless IMDs. In [47], the authors describe the combination of CMOS with a planar ferromagnetic material to device Hall and flux-gate magnetic field sensors, whereas [48] presents a two-dimensional vector magnetic sensor comprised of CMOS technology with ferromagnetic materials. On the other hand, [49] reported results of on-chip inductor fabrication in CMOS technology with magnetic material. Reference [50] presented a review on measuring resonator’s Q-factor integrated with analog and mixed-signal circuits. A navigation system for 3D localization during surgeries has been described in [51], which includes circuitry to wirelessly transfer power and data. Our prior works ([52], [53]) described the fabrication of hybrid ME antennas capable of sensing a wide range of magnetic fields (picoTesla to miliTesla range), and they identified the importance of operation with dual frequency mode for neural implantable devices.

Building on past explorations, this paper includes first proof-of-concept results from a prototyping measurement platform utilizing a dual-mode miniaturized hybrid ME antenna-sensor in a bidirectional wireless link to perform efficient concurrent magnetic field sensing and energy harvesting at two different frequencies. Furthermore, to provide a foundation for circuit simulations, a hybrid ME antenna circuit model exhibiting two different resonance frequencies has been created and verified through measurement results with sensing and energy harvesting circuitry, which demonstrates the integration of an ME antenna-sensor and CMOS circuits with negligible loading effects. An introduction to the standalone energy harvesting capability of an ME antenna and the free-standing sensing capability of an ME sensor have been provided in [54], [55], [56] utilizing a wired platform to explore the CMOS integration with the magnetoelectric material. On the contrary, in this work, an RF measurement platform has been optimized to evaluate integrated functionalities of wireless power transfer, low-frequency magnetic modulation and wireless sensing utilizing a hybrid dual-mode ME antenna and an SDR as wireless transceiver. Fig. 1 displays a visualization of the hybrid ME antenna, in which the 2.57 GHz resonance model for energy harvesting and 63.63 MHz resonance model for sensing represent a single ME antenna with dual resonance modes in the circuit domain. As depicted in Fig. 1, the ME antenna receives wireless power from an external transceiver to initiate the energy harvesting operation using its high-frequency resonance mode. It is envisioned that this energy harvester will supply power to the implant that will perform the low-frequency neural signal sensing and magnetic modulation. Furthermore, the results are the first from experiments using waveforms with the shape of magnetically modulated action potentials to emulate neuronal magnetic fields (NMFs) that occur due to neuronal activity. In addition, the sensitivity of the wireless platform to enable the low-frequency magnetic modulation has been experimentally validated by determining the limit of detection (LoD), which substantiates the magnetic modulation of low-frequency signals. It is important to note that, even though the hybrid ME antenna and sensor can sense sub-picoTesla magnetic fields ([52], [53]), the SDR can reconstruct an original time-domain signal residing in the low microTesla range. The limit of detection with the SDR as the external transceiver is elaborated in Section VI-B based on measurements.

The paper is organized as follows: Section II provides background information on ME antenna characteristics and power transfer efficiency (PTE) measurements for wireless energy harvesting. Section III presents the standalone circuit simulation and measurement results associated with the high-frequency ME antenna model. Sections IV and V describe the energy harvester and its incorporation into the high-frequency ME antenna modeling based on experimental measurements. Section VI combines the simultaneous energy harvesting and sensing circuit models, and it contains validation measurements with dual-mode operation of magnetic sensing with different signals, including synthesized neural action potentials. Section VII provides the conclusion.

## II. BACKGROUND

### A. CHARACTERISTICS OF ULTRA-COMPACT DUAL-MODE ME ANTENNAS

**FIGURE 2.**
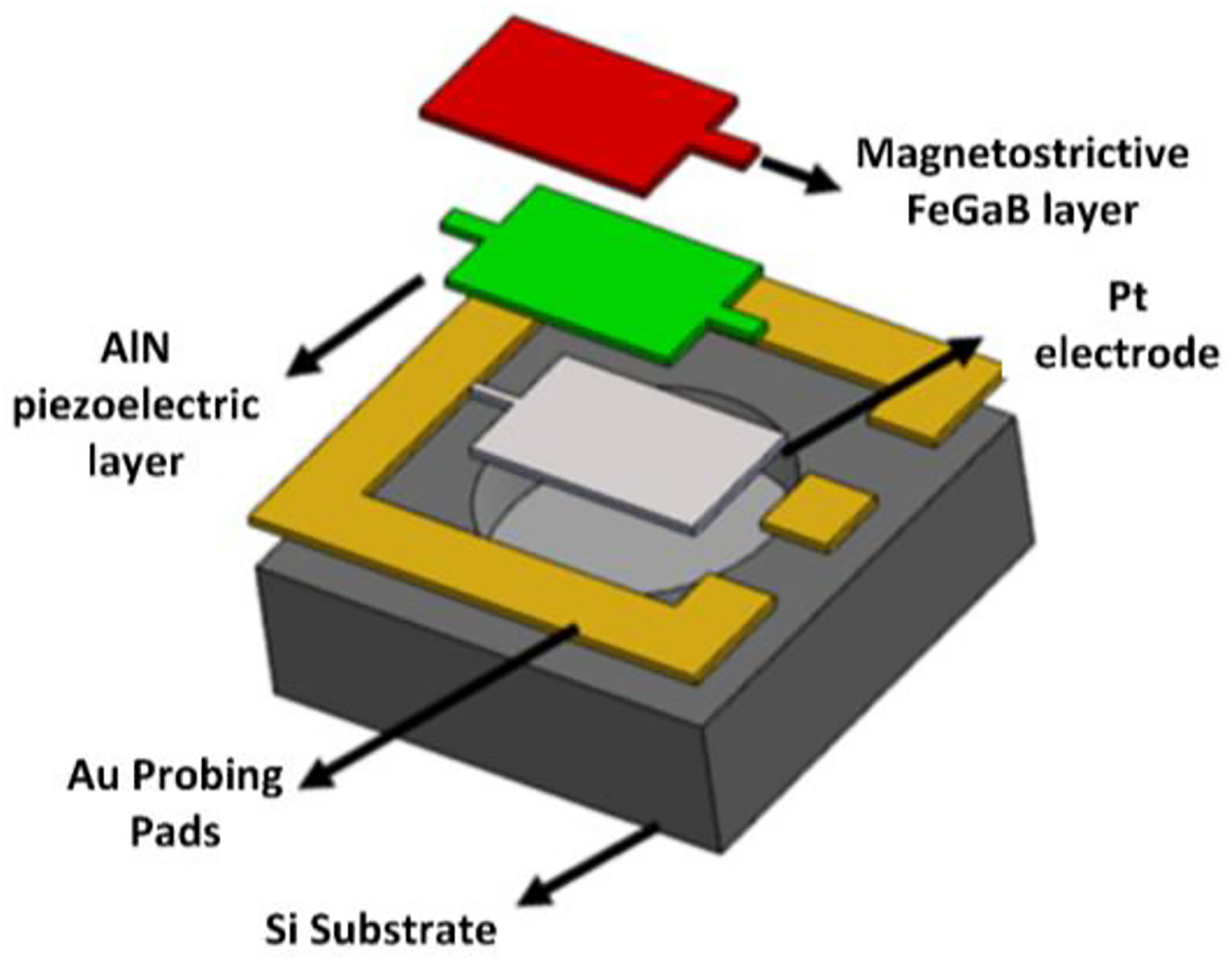
Construction of a hybrid mode ME device.

The need for sensing sub-nano Tesla magnetic fields from neuronal activity is identified in [57]. The integration of magnetic materials with CMOS technologies creates opportunities for a versatile range of biomedical applications by the virtue of dimensional scaling properties. As part of this research, a small ME antenna-sensor component has been devised using multiferroic materials with the advantage of multiple ferroics properties and a capability of sensing magnetic fields in the picoTesla to miliTesla range [52], [53]. Fig. 2 shows the construction of a hybrid mode ME resonator with its layers to fabricate a mechanically coupled piezoelectric (Aluminium Nitride, AIN) and magnetostrictive (Iron Gallium Boron, FeGaB) heterostructure. A piezoelectric and magnetostrictive heterojunction facilitates the benefit of ferroics: the piezoelectric thin-film merges electrical polarization and mechanical strain, whereas the magnetostrictive material incorporates magnetic polarization and mechanical strain [52]. The combination of the aforementioned ferroics properties enables a single ME antenna to transmit (TX) and receive (RX) electromagnetic (EM) waves at different frequencies due to the different strain levels induced by magnetic fields. In RX mode, an incoming EM wave produces a strain in the magnetic layer that is transferred to the piezoelectric layer to generate a radio frequency (RF) voltage across the thickness, which can be utilized for efficient wireless power transfer for energy harvesting at GHz frequencies. On the other hand, the TX mode of the ME device exhibits the property of an ultra-sensitive magnetic sensor capable of sensing very small magnetic fields that reside at low frequencies, which occurs through transferring mechanical strain in the magnetic layers while the piezo layer is exposed to an AC magnetic field and DC bias. Therefore, the dual frequency mode functionality qualifies the ME antenna as a promising candidate for battery-less neural sensors realized as IMDs. Fig. 2 displays the 3D structure of the heterojunction used to fabricate the ME resonator in this work. The hybrid ME device exhibiting two different frequencies is fabricated by utilizing three of such rectangular ME resonators in parallel. These three parallel ME resonators are connected to a gold ground ring with ground-signal-ground (GSG) pads. The use of several parallel resonators result in better performance of the ME device, but with increased overall size. A combination of three resonators has been chosen to obtain sufficiently high performance for neural implantable devices. The size of the individual resonator is 250 × 50 *μ*m^2^, resulting an overall ME antenna size of 250 × 174 *μ*m^2^ with three parallel resonators, including gaps between the resonators. The thickness of the combined AlN and FeGaB layers is 1 *μ*m, each comprising 500 nm. The Platinum (Pt) layer in Fig. 2 is 50 nm thick. Finally, the ME resonator is etched into the Silicon (Si) substrate that provides protection against damping, leading to improved performance. The resonance frequencies of the rectangular ME elements are defined by the size (thickness and width) of the AlN/FeGaB heterostructure-based piezoelectric thin-film. The corresponding resonance frequencies across width and thickness of the piezoelectric thin-film are determined by $f_{\text{width}}=\left(\frac{1}{2\cdot W_{0}}\right)\sqrt{\frac{E}{\rho }}$ and $f_{\text{thickness}}=\left(\frac{1}{2\cdot t}\right)\sqrt{\frac{E}{\rho }}$, where W_0_ and *t* are the width and thickness of the piezoelectric thin-film respectively, E is the equivalent Young’s modulus, and *ρ* equivalent density of the resonator stack. The thickness of the piezoelectric thin-film is much smaller compared to its width. Thus, the thickness mode resonance frequency of the ME device is significantly higher than the width mode resonance frequency, which is why the thickness mode resonance frequency has been utilized for wireless energy harvesting and the width mode resonance frequency is well-suited to sense low-frequency neural signals. Furthermore, the ample difference of the resonance frequencies avoids any significant interference across the frequency bands during simultaneous operation. This paper shares new findings that demonstrate the characteristics of a dual-mode ME device, which lead to the described development of a bidirectional wireless link to assess the simultaneous sensing and energy harvesting capabilities.

### B. POWER TRANSFER EFFICIENCY OF A STANDALONE ME ANTENNA

Wireless power transfer is an important consideration during the design and fabrication of IMDs to avoid multiple invasive surgeries to replace batteries with limited lifespan. Electromagnetic, ultra-sound and acoustic mediums are predominantly considered for wireless power transfer to IMDs [23], [24], [58]. The need of aggressive miniaturization of antennas and circuits limits the wireless power transfer for energy harvesting within implants. References [59] and [60] have shown that low gigahertz frequencies are well-suited for wireless power transfer to millimeter-sized implantable devices. Reference [61] presented a mm-size neural implant utilizing magnetoelectric effects for highly efficient power and data transfer, and [62] exhibits a wireless millimetric magnetoelectric implant to receive power and data for peripheral nerve stimulation.

**FIGURE 3.**
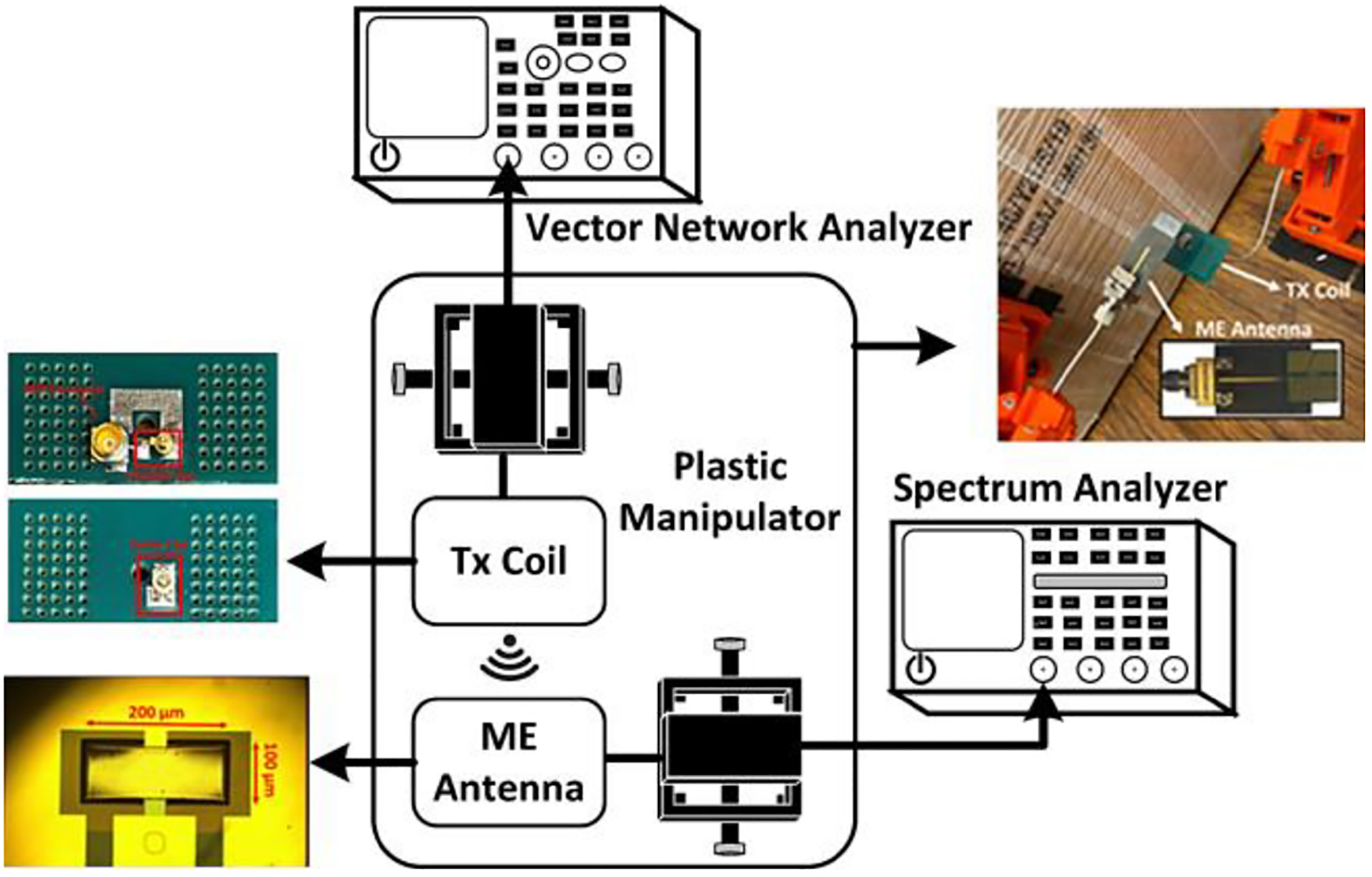
Power transfer efficiency measurement setup.

**FIGURE 4.**
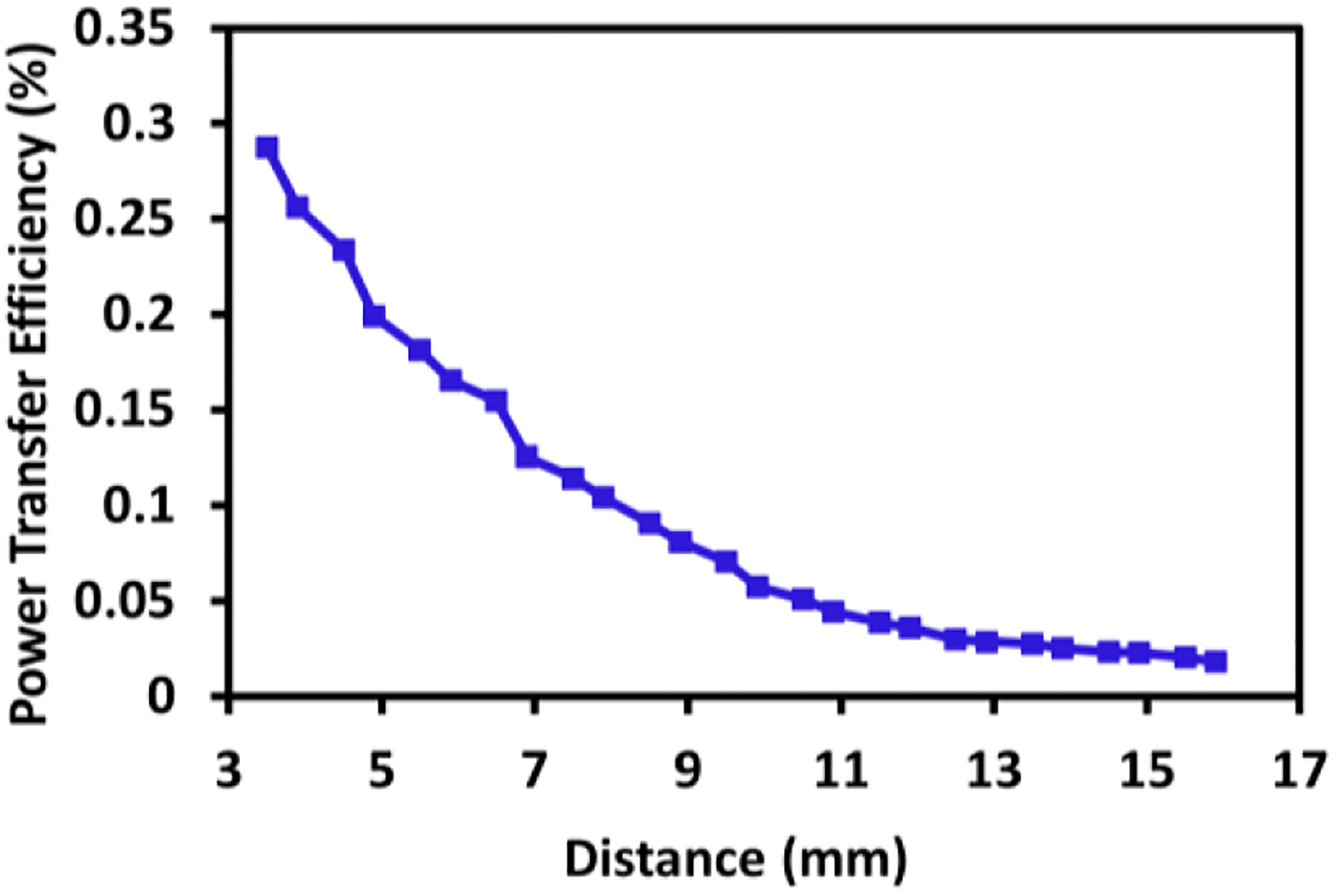
Measured PTE for the TX coil and ME antenna.

Fig. 3 shows the experimental setup for the power transfer PTE measurement of an ME antenna and a TX coil at the high resonance frequency of 2.57 GHz. A single-turn TX coil was fabricated on an FR4 PCB with a capacitive L-matching network to properly align its resonance frequency to the ME antenna’s high resonance frequency mode for maximum power transfer. The design of the 10 mm × 10 mm TX coil is reported in ([60], [63]) together with a discussion about the choice of this wireless link for highly efficient power transfer while preserving the advantage of on-silicon sub-mm device sizes. The PTE varies linearly with the change in magnetic flux density to which the ME antenna is sensitive to. Considering the fact that the ME antenna will change its orientation after initial placement, the TX coil has been designed as a one-turn rectangular coil comprising trace lines along X and Y axes, which produces magnetic flux along the X and Y directions. Due to the one-turn loop, the magnetic fluxes generated by copper traces constructively interfere at the center of the coil, producing a magnetic field along the Z axis. Therefore, the ME antenna receives power from all directions in this design. PTE is defined as the ratio of received power (by the ME antenna) over a wireless link to the transmitted power, considering the loss in received power due to PCB traces in ME antenna. In order to estimate PTE over the air, the ME antenna is connected to an Agilent E4446A PSA spectrum analyzer (SA) to measure the received power, whereas the TX coil is powered by a Keysight N9320B vector network analyzer (VNA) set to 7 dBm output power, which is also used to measure the impedance. A magnetic strain will be induced in the magnetostrictive layer of the ME antenna upon receiving an RF signal. The strain will be further transferred to the piezoelectric material layer, resulting in a production of an RF voltage as discussed in Section II-A. The generated RF voltage can be used to power the CMOS circuits at the 2.57 GHz resonance frequency [63]. Fig. 4 displays the plot of PTE vs. distance between the TX coil and ME antenna from the measurement. Two plastic manipulators with a spatial resolution of less than 200 *μ*m were used in order to arrange and control the position in the x, y, z directions of the ME antenna and the TX coil without hampering the electromagnetic field strength at the time of measurements. Reference [51] recently reported 3D surgical alignment with 100 *μ*m resolution. It is evident from Fig. 4 that the highest PTE is achieved by reducing the distance between the TX coil and ME antenna during the measurement, of which the results are described in Sections V and VI. Table 1 compares the dual-mode ME antenna’s PTE with several other state-of-the-art single-mode antennas. It can be observed that there is a trend of improved efficiency with increasing dimensions. Despite of its small size, the dual-mode antenna has a suitable efficiency compared to other reported results.

### C. ME ANTENNA CHARACTERIZATION TOGETHER WITH A WIRELESS ENERGY HARVESTER

**FIGURE 5.**
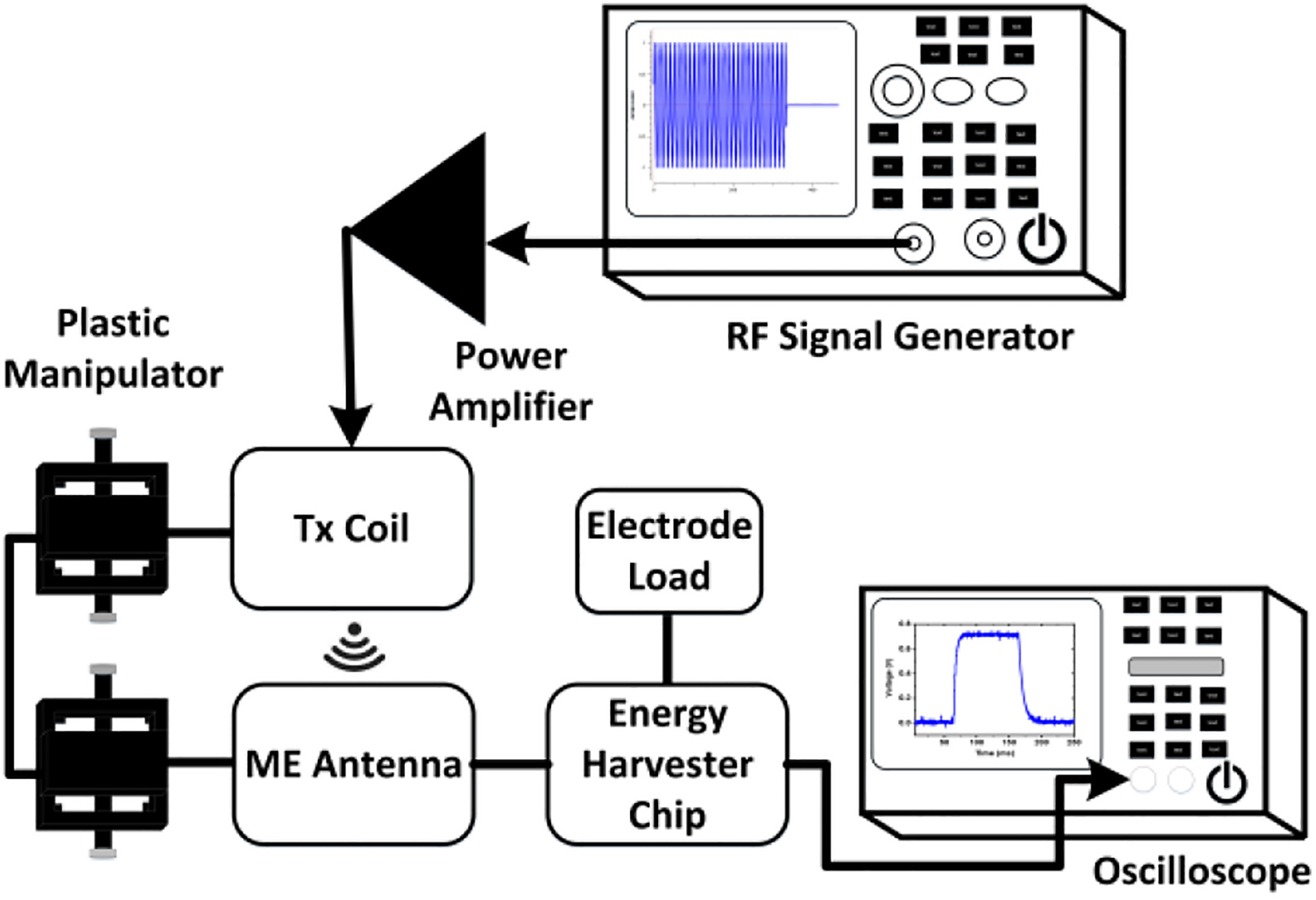
Experimental test setup for wireless energy harvesting with an ME antenna and electrode load.

Fig. 5 displays the experimental setup to evaluate the wireless energy harvesting operation. This ongoing integrated system development effort aims to utilize the energy harvester as part of a neural stimulation device in which electrical pulses are applied through electrodes [69]. The approach aims to develop a neural implantable device that maintains high achievable PTE by keeping the specific absorption rate (SAR) within limits imposed by the Federal Communications Commission (FCC). The SAR simulation is beyond the scope of this paper due to its strong dependence on the application and use cases. However, the transmitted power can be duty-cycled to maintain high PTE within the SAR limit. The research in [52] investigated the PTE in tissue, where measurements showed an approximately three times higher value compared to air. The phenomenon occurred as a result of the high relative permittivity of the tissue compared to the air medium at 2.57 GHz, resulting in shorter wavelength. Hence, due to the shorter wavelength, the EM wave can propagate near the TX coil, which improves the PTE. In this experimental setup, an RF signal generator (Siglent SSG3032X) produces the pulse-modulated signal with a power of −12 dBm. For the subsequent simultaneous wireless sensing and energy harvesting measurements, the signal generator was replaced with the USRP B200mini-i SDR as an external transceiver. In Fig. 5, the pulse-modulated signal from the signal generator is applied to a HPA3000 power amplifier with a gain of 45 dB to excite the TX coil. The TX coil then initiates the wireless power transmission at the high-frequency resonance mode of the ME antenna (2.57 GHz). This power is wirelessly transferred from the TX coil to the ME antenna as shown in Fig. 5, and the output power of the ME antenna is directly measured with a SA. The distance between the TX coil and ME antenna is approximately 15 mm. This condition resulted in a measured power of −1 dBm output power of ME antenna, which becomes the input signal power to the energy harvester chip. As mentioned in Section II-A, the ME resonators are connected to GSG pads that are wire-bonded to a PCB with an SMA connector. A loss of 35% of the wirelessly received power due the effects on the PCB has been considered during the PTE calculation, which leads to an overall PTE of 0.028% at a distance of 15 mm between the TX coil and ME antenna. Moreover, the reduction of the power from TX coil to received power by ME antenna is caused primarily by the path loss between the TX coil and ME antenna. Furthermore, the plastic manipulators and cables contribute to this loss. An oscilloscope was used to measure the transient response (voltage vs. time) at the energy harvester output. The measurement results are presented in Section V for comparison with the simulation results based on the antenna model.

## III. BEHAVIORAL CIRCUIT MODEL FOR ENERGY HARVESTING CHARACTERISTICS OF ME ANTENNAS

### A. MODIFIED BUTTERWORTH VAN-DYKE MODEL

**FIGURE 6.**
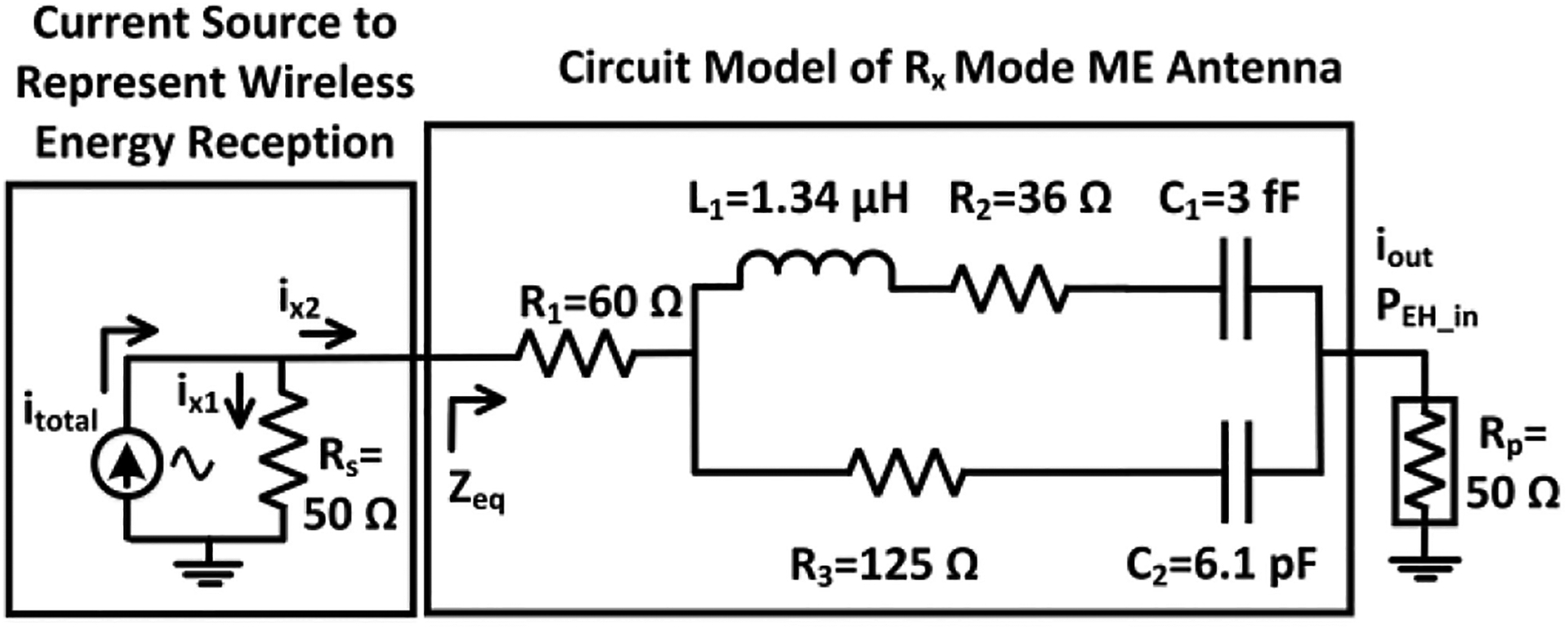
RX-mode ME antenna circuit model for wireless power transfer and energy harvesting.

The Modified Butterworth Van-Dyke (MBVD) model can be used to analyze the behavior of magnetic resonators [70]. It forms the fundamental basis for representing an ME antenna in the circuit domain, as displayed in Fig. 6. In [71], the AlN-based film bulk acoustic wave resonator (FBAR) has been modeled and characterized using an MBVD model. References [72], [73], [74] further describe the modeling of magnetic resonators, where FBAR thin films utilize the admittance of the width and thickness mode of the ME resonator, which can be fitted with an MBVD model to extract electromechanical parameters such as the electromechanical coupling coefficient k_t_^2^ and the quality (Q) factor. In this work, we followed a similar approach as in [70] to extract the MBVD model parameters from the FBAR resonator visualized in Fig. 2. In Fig. 6, the electrical components include C_2_, which is the device capacitance determined by the device geometry. The electrical resistance R_3_ is associated with the dielectric loss. On the other hand, C_1_ (motional capacitance), L_1_ (motional inductance) and R_2_ (motional resistance) are the components that model mechanical characteristics. They can be analytically expressed as $R_{2}=\frac{1}{\omega^{2}C_{\_2}k_{t}^{2}Q}$, $C_{1}=\frac{8}{\pi^{2}\left(C_{\_2}k_{t}^{2}\right)}$, and $L_{1}=\frac{1}{\omega^{2}C_{1}}$. The series resistance R_1_ is connected to both branches as the electrical loss of the electrode(s). At the resonance frequency (*ω*), C_1_ and L_1_ cancel each other. The k_t_^2^ coupling coefficient represents the efficiency of the electrical and acoustic energy conversion, and the Q-factor defines the ratio of the energy stored in the vibrating resonant structure to the energy dissipated (per cycle) by the damping processes. Modeling of the ME antenna allows to estimate its performance while exposed to wirelessly received power during the excitation of an energy harvesting circuit. In particular, the measured output power of the ME antenna, expressed as *P*_*EH*_*in*_, can be verified with transient simulations using a conventional circuit simulator. The model allows to optimize the interface circuits based on the extracted resonator impedance characteristics. In Fig. 6, the values of the ideal components (*R*_1_*, R*_2_*, R*_3_*, L*_1_*, C*_1_*, C*_2_), of this RX-mode ME antenna circuit model were extracted at a resonance frequency of 2.57 GHz by measuring its S-parameters as in [70]. From the measurement results, the component values in the model were calculated using the curve fitting command in MATLAB with a defined error goal of 1%.

### B. PROPOSED ME ANTENNA MODEL

As indicated in Fig. 6, the wirelessly received power has been modeled through the injection of a current at the input of the MBVD circuit model. Modeling of the wirelessly received power during circuit simulation is necessary to characterize the energy harvesting operation together with ME antenna circuit model in the design phase. Here, the wirelessly received power is a pulse-modulated signal as described in SectionV. It is expected that the ME antenna is terminated by 50 Ω directly or through a matching network, which is why a 50 Ω resistor (*R*_*S*_) has been included in the model together with the ideal current source. Note that the ME antenna was also terminated with a 50 Ω port (*R*_*p*_) during the measurement of its S-parameters for the MBVD model extraction. From Fig. 6, it can be seen that *R*_*s*_ and the equivalent impedance (*Z*_*eq*_) of the MBVD model form a current divider, where *i*_*x*2_ is the current through the MBVD circuit, which is equal to *i*_*out*_. Analytically, *P*_*EH*_*in*_ can be calculated from the root-means-square (*rms*) value (*i*_*rms*_*out*_) of *i*_*out*_. Based on the current division property of the circuit, *i*_*x*2_ can be expressed as follows:

(1) $$i_{\text{out}}=i_{x2}=\frac{R_{s}}{R_{s}+Z_{eq}}i_{\text{total}}$$

where:

(2) $$Z_{eq}=R_{1}+\left\{\left(j\omega L_{1}+R_{2}+\frac{1}{j\omega C_{1}}\right)∥\left(R_{3}+\frac{1}{j\omega C_{2}}\right)\right\}+R_{p}$$

**FIGURE 7.**
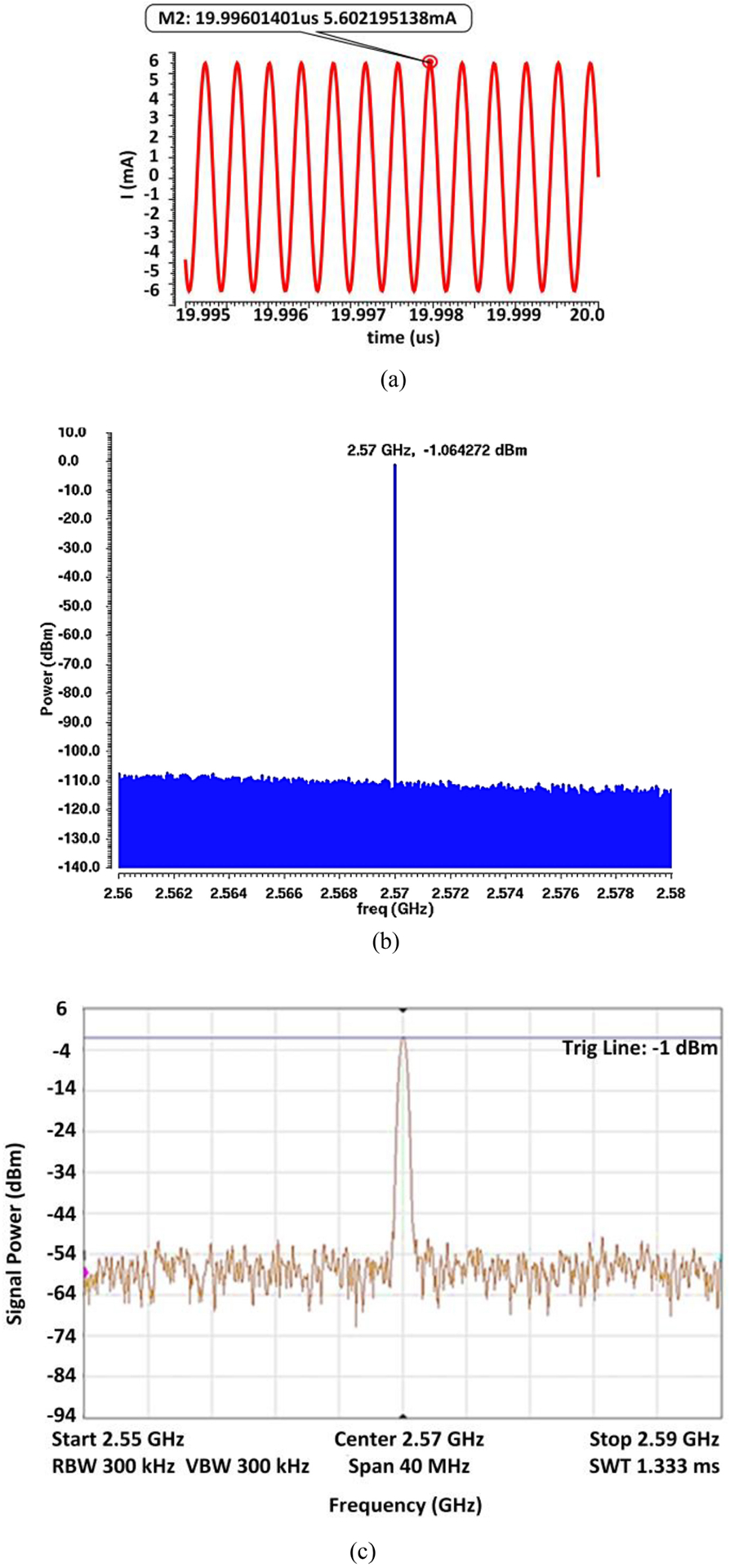
ME antenna characterization: (a) simulated peak output current at the ME antenna circuit model output, (b) simulated output power spectrum, (c) spectrum analyzer image of the measured output power of the ME antenna.

*Z*_eq_ can be found as 235.7 Ω at the ME resonance frequency of *f* = *ω*/(*2π*) = 2.57 GHz with the model component values annotated in Fig. 6. The magnitude of *itotal* has been selected as 32 mA such that the model produces −1 dBm of power at the output as in the characterization measurement with the setup described in Section II-C. From Fig. 7(a), it can be seen that the peak output current from transient simulation is 5.6 mA (3.96 mA *rms*) and converted into equivalent output power in dBm with 50 Ω impedance matching condition.

Fig. 7(b) displays the simulated output power spectrum of the MBVD circuit model, showing the −1.06 dBm component at the resonance frequency. This value agrees with the measured ME antenna output power in Fig. 7(c). The next section includes an evaluation of the standalone energy harvester performance with electrode load using a wired configuration to determine its efficiency. Section V describes simulation results of the ME antenna model with an energy harvesting circuit, which are compared to chip measurement results for validation.

## IV. CASE STUDY: ENERGY HARVESTER CIRCUIT DESIGN CONSIDERATIONS

**FIGURE 8.**
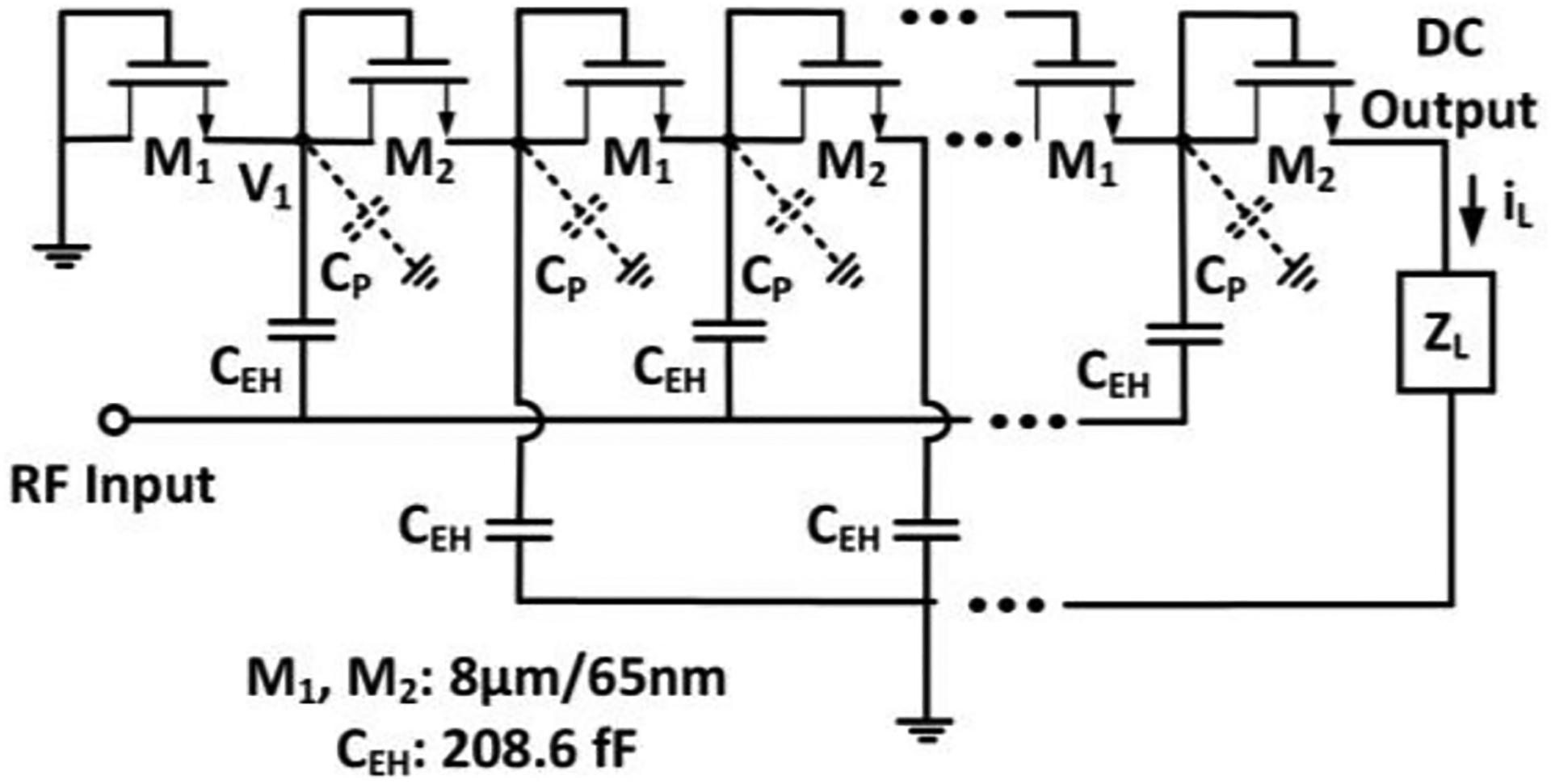
Simplified schematic of the multi-stage rectifier for energy harvesting.

CMOS-based rectifier topologies, such as the conventional Dickson rectifier [75] or the cross-connected differential topology [76], are widely used in modern wireless IMDs to inject charges to the tissue from scavenging the incoming RF energy [77], [78], [79]. Fig. 8 shows the typical Dickson rectifier that consists of two pumping transistors (M_1_, M_2_) and a coupling capacitor (C_EH_) in each stage. When the RF input is negative, then M_1_ is forward-biased if the input amplitude is higher than the threshold voltage of M_1_, such that the DC node at the junction of M_1_ and M_2_ voltage (V_1_) is slowly charged up whenever M_1_ conducts. Low-threshold diodes or transistors are helpful when the input swing is limited. During the positive cycle, M_2_ will start conducting when the voltage difference between V_1_ and the output voltage becomes higher than the threshold voltage, and the charge accumulated on C_EH_ during the previous cycle is dumped to C_EH_ connected on the other side of M_2_ until there is no voltage difference. Therefore, by stacking a multiple of N stages, the output voltage formula has been derived in [75] as:

(3) $$v_{\text{OUT}}=N\times \left(\frac{C_{EH}}{C_{EH}+C_{P}}\times v_{IN}-\frac{i_{L}}{f\cdot \left(C_{EH}+C_{P}\right)}-V_{th}\right)-V_{\text{th}}$$

In the above equation, v_IN_ is the input RF signal swing defined as the peak-to-peak voltage of the signal, C_EH_ is the coupling capacitor between the RF input port and each pumping transistors, C_P_ is the parasitic capacitance at each node, i_L_ is the load current shown in Fig. 8, *f* is the frequency of the incoming RF signal, and V_th_ is the threshold voltage of the transistors. From this equation, we can intuitively conclude that the output voltage depends on the input voltage swing, load condition, number of stages (N) and the intrinsic characteristics of the pumping transistors.

The Dickson topology has been well explored and improved by many works to expand the usage scenarios. The efforts in [80], [81] introduced gate biasing techniques to help pumping transistors conduct more efficiently under ultra-low-power conditions. In [82], [83], [84], the authors adopted reconfigurable control of the number of stages to improve the optimal input power range such that the design can adapt to the changing input power conditions. The work in [85] provides mathematical insights into the behavior of the rectifier to better guide the design decisions related to the choices of transistor dimensions, the number of stages and the optional compensation voltage.

A key challenge in IMD applications is to realize high efficiency and the capability to inject sufficient amount of charge during a certain time window while maintaining a high integration level for all required components at the same time. Miniaturization implies low-quality factor on-chip passive components (e.g., inductors and capacitors), causing more power loss at the interface. Hence, the inductance and capacitance should be carefully selected and implemented to avoid efficiency reduction and excessive layout area. Considering these constraints, we designed a 10-stage conventional Dickson rectifier that was fabricated in a 65nm CMOS process with dimensions of 400 *μ*m × 75 *μ*m. With regards to the schematic in Fig. 8, C_EH_ are metal-insulator-metal (MIM) capacitors, and the transistors (M_1_, M_2_) are low-threshold NMOS devices with minimum length. Assuming perfect and lossless matching conditions, the input amplitude at this power level seen at the rectifier is 0.691 V, which is higher than the threshold voltage of the transistor. A detailed description of the fully integrated energy harvester chip can be found in [86]. It is noteworthy that designing a multistage energy harvester at high frequencies is challenging due to the efficiency reduction. Increasing the frequency leads to higher dynamic power dissipation, and thus to reduced efficiency. For example, [87] includes a 3-stage DC-to-DC converter with 10 MHz clock frequency and 49% efficiency, whereas [88] reported a 46% efficiency with a 4 MHz pump frequency. Since the ME antenna exhibits a high-frequency mode for wireless energy harvesting at 2.57 GHz, the presented energy harvester utilizes a 10-stage Dickson rectifier with 9.5% efficiency. The input impedance of the 10-stage rectifier at a −3 dBm power level is 81.52 – j470.95 Ω based on large-signal S-parameter (LSSP) analysis with harmonic balance simulation in Cadence Spectre. In comparison, [89] includes a 4-stage differential topology designed at 2.4 GHz frequency with 15% efficiency at 0 dBm power. Furthermore, [90] reports a 3-stage differential topology with 47.1% efficiency for a −6 dBm input at 2.4 GHz, but requires to be highly optimized for a certain load in order to realize high-efficiency operation. Multiple works in this frequency range utilize high-Q off-chip matching networks to boost the efficiency [91], but in this work the rectifier is a fully integrated circuit to minimize size for implantable applications.

**FIGURE 9.**
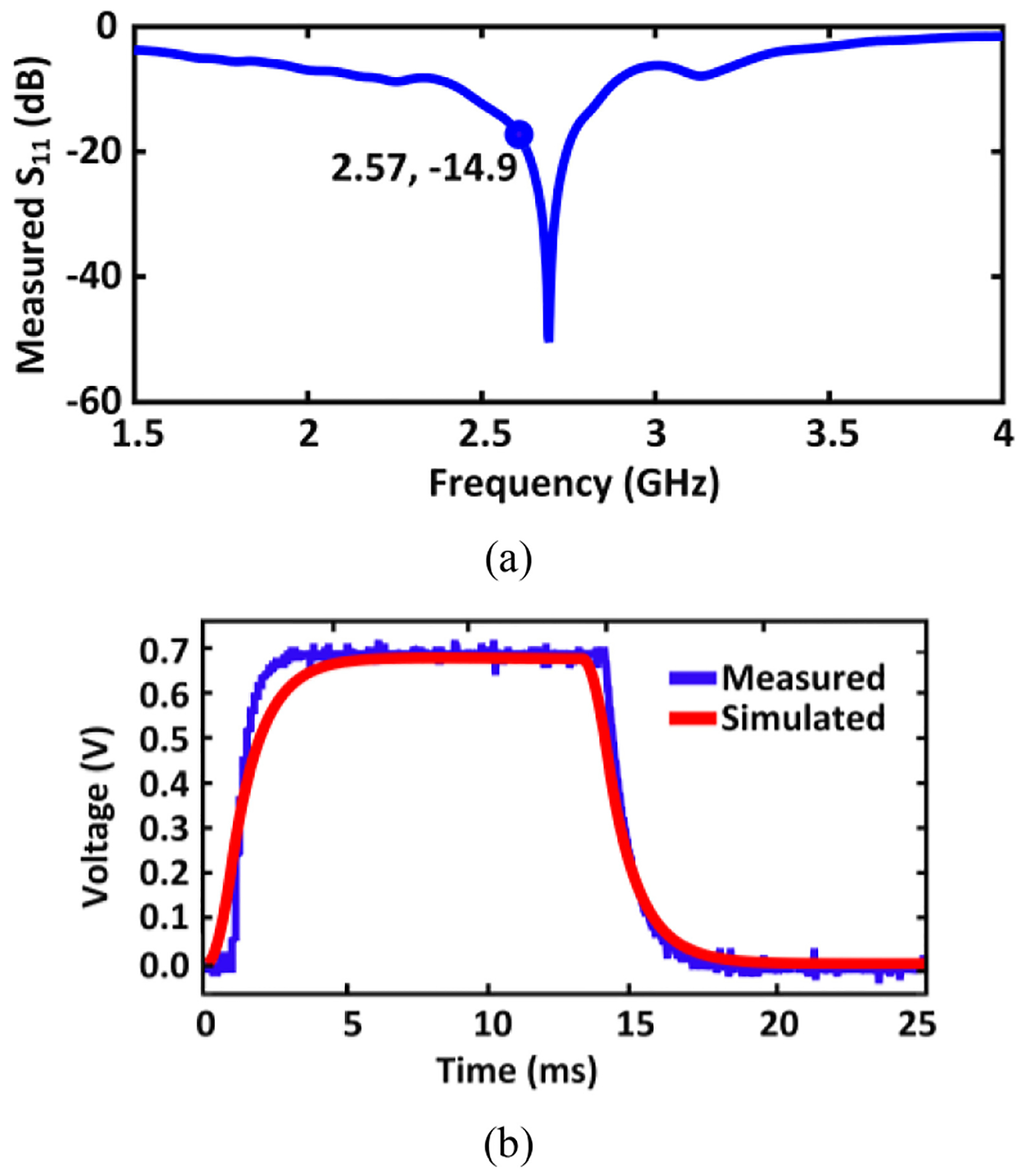
Wired characterization measurements of the standalone energy harvester: (a) measured S_11_ with matching network, (b) measured and simulated output voltage with only energy harvester with electric load.

**FIGURE 10.**
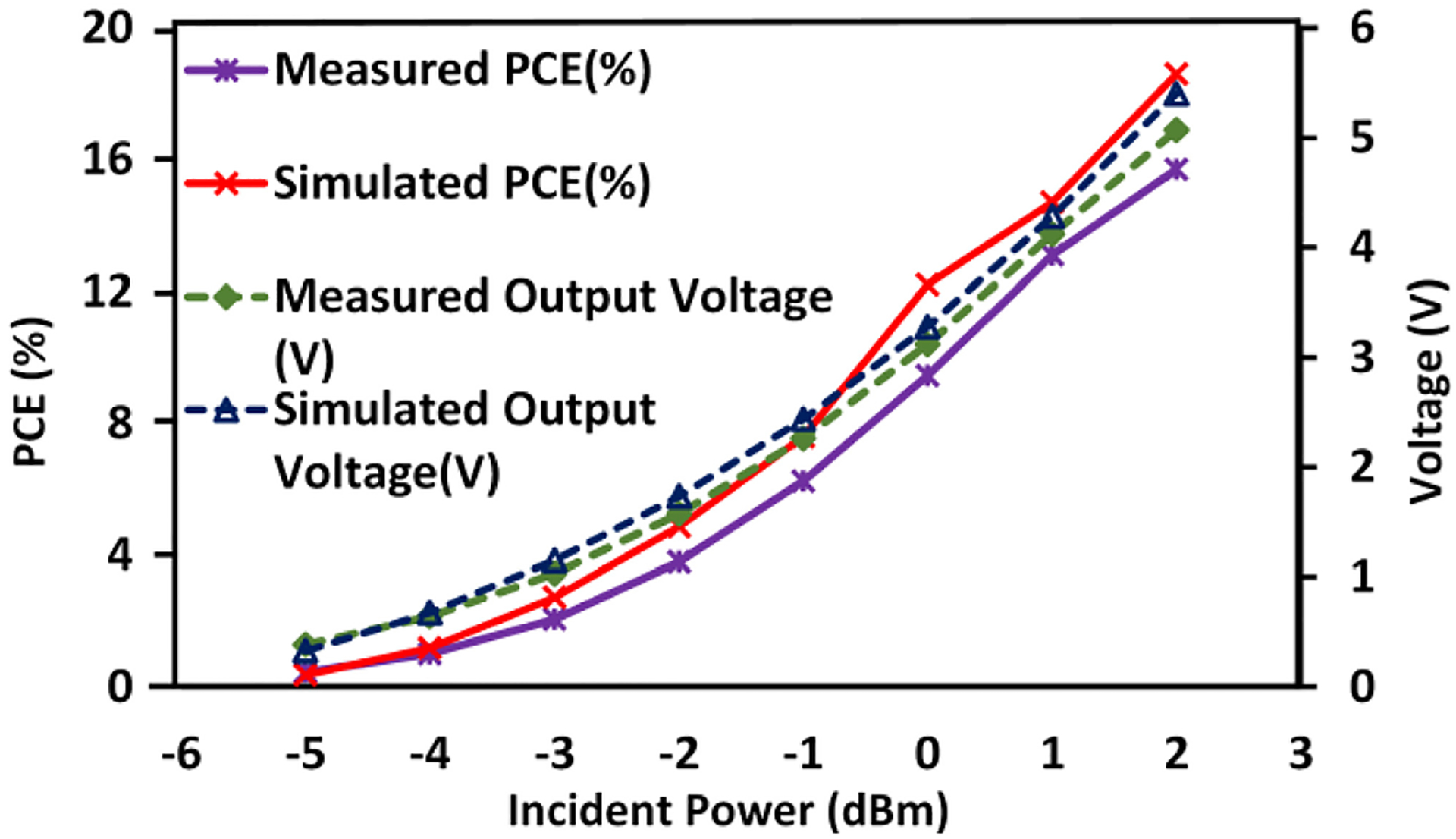
Measured energy harvester PCE and output voltage with electrode load, and its simulated PCE and output voltage with the corresponding electrode model.

**FIGURE 11.**
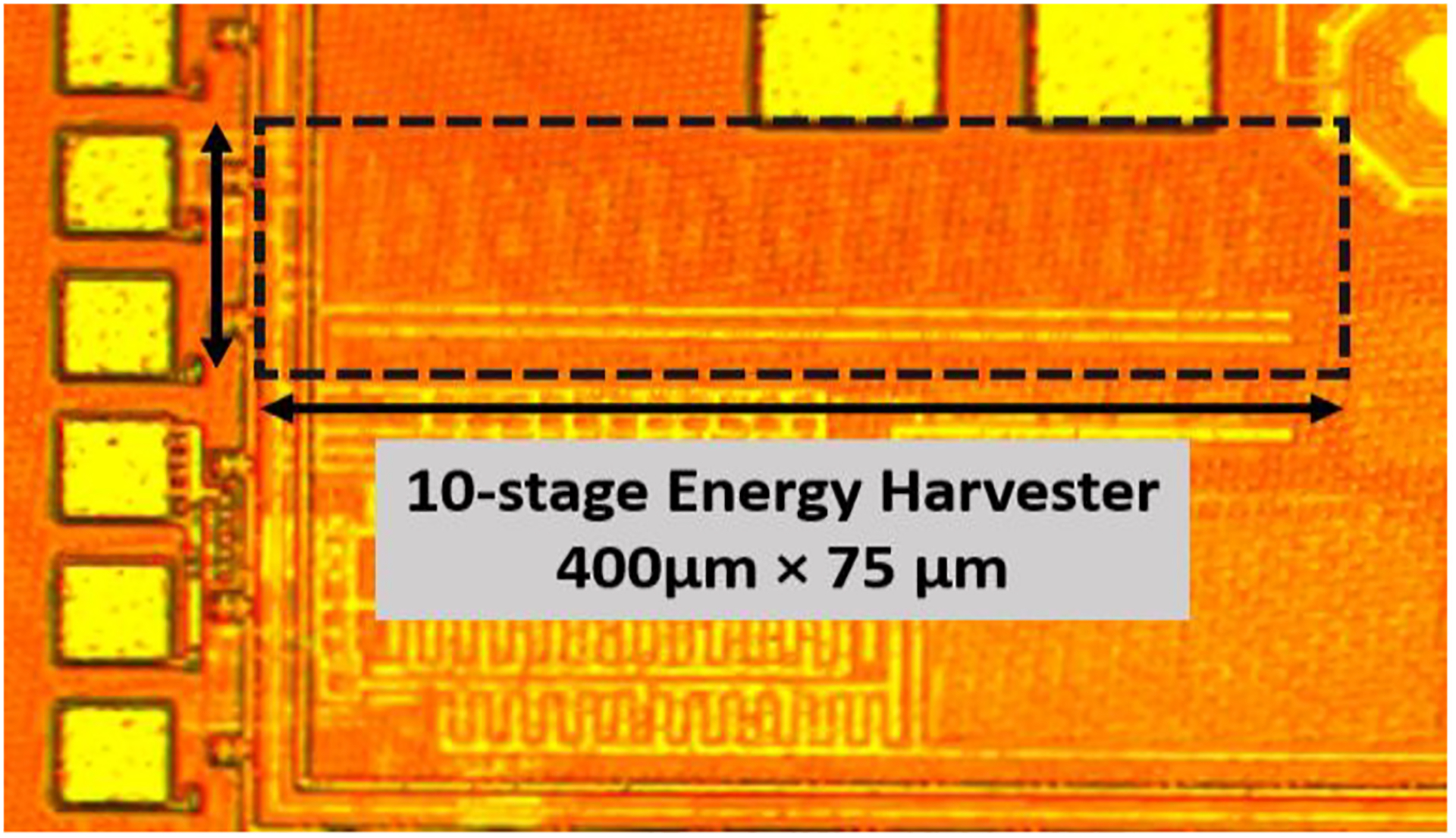
Micrograph of the die with the energy harvester.

Fig. 9(a) displays S_11_ versus frequency measured at 2.57 GHz. We have measured the standalone energy harvester with electrode load using different input power levels ranging from −5 dBm to −1 dBm, which resulted in negligible S_11_ differences of less than 0.2 dB. The circuit model of the electrode load is discussed in Section V. It can be observed that S_11_ is −14.9dB at the 2.57 GHz frequency of interest, such that only 3.4% of input power is reflected. Fig. 9(b) includes the simulation and wired measurement results of the standalone energy harvester output voltage with electrode load. The measured output voltage with −3 dBm input power is 710 mV, and the simulated output voltage reaches 702 mV. The key metrics from the measurements are summarized in Table 2. The power conversion efficiency (PCE) is often used to quantify how efficiently an energy harvester operates, and it is defined as the ratio of the output power consumed by the load (P_out_) to the incident power received by the antenna (P_in_) [92], [93]. Hence, the PCE can be regarded as end-to-end PCE, which includes the reflection loss of the impedance mismatch between the antenna and the matching network, as well as the rectifier efficiency itself. Fig. 10 show the measured and simulated end-to-end PCE and output voltage with electrode load. The measurement results are in a good agreement with the pre-layout simulation results, having acceptable errors due to parasitics. During the system-level wireless power transfer measurements (Sections V and VI), there are 2 dB more losses at the antenna interface compared to the standalone wired energy harvester test setup, which is why a correspondingly higher power level has been applied during the system level measurements. Fig. 11. displays the die photo of this energy harvester.

## V. EXPERIMENTAL MODEL VERIFICATION WITH AN ENERGY HARVESTER INTERFACE

**FIGURE 12.**
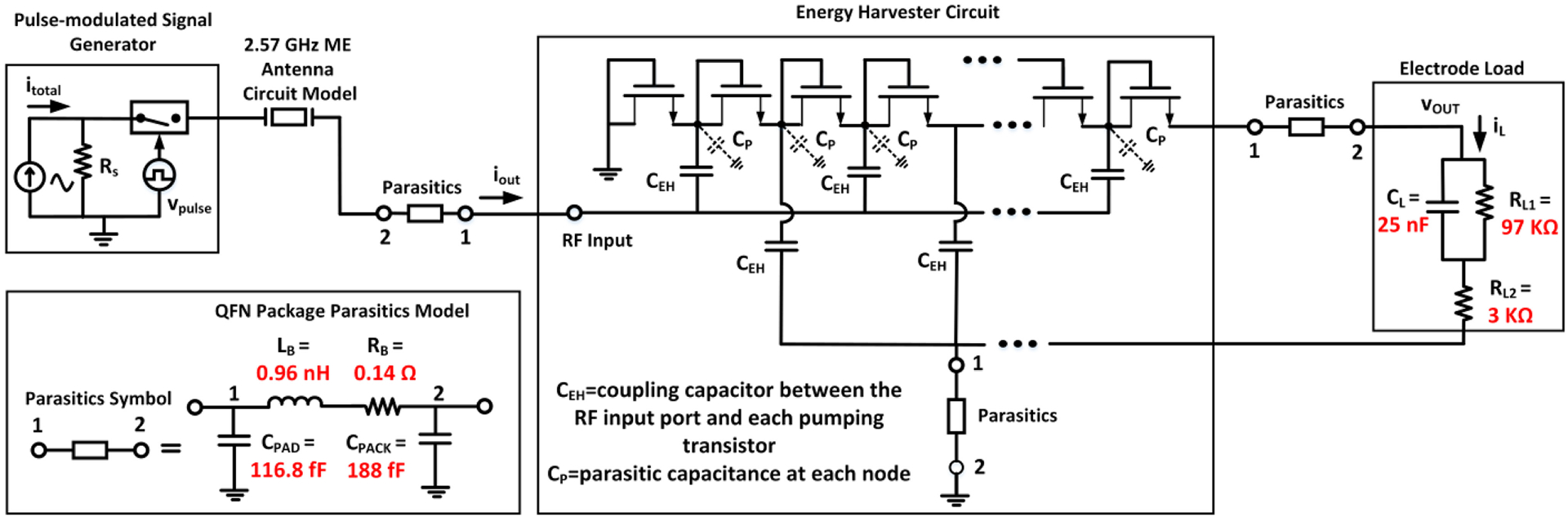
Combined circuit model of the ME antenna and the energy harvester (simplified schematic) with electrode load.

Fig. 12 displays the circuit model of the ME antenna together with the energy harvester from Fig. 8. To emulate pulse modulation, a behavioral switch controlled by a stream of control pulses was included in the signal generation block. Hence, the simulated antenna excitation yields the same response as the pulse-modulated RF signal from the TX coil within the measurement setup described in Section II-C. For comparison, a control signal (v_pulse_) with 15 ms pulse width was applied to modulate the current source at the input of the ME antenna circuit model, producing pulsed output power to initiate the energy harvesting operation during transient simulations with the Spectre Cadence simulator. The output of the energy harvester is connected to an electrode load as shown in Fig. 12; in which C_L_, R_L1_ and R_L2_ are specified as 25 nF, 97 KΩ and 3 KΩ, respectively. This electrode model was selected based on commonly used electrodes for neural stimulation [94]. The associated package parasitics (C_PACK_), bonding wire (L_B_, R_B_) and pad capacitance (C_PAD_) of the QFN-packaged 65nm CMOS die were included during the simulations. L_B_, R_B_, C_PAD_ and C_PACK_ in Fig. 12 were modeled as 0.9596 nH, 0.14 Ω, 116.8 fF and 188 fF, respectively.

**FIGURE 13.**
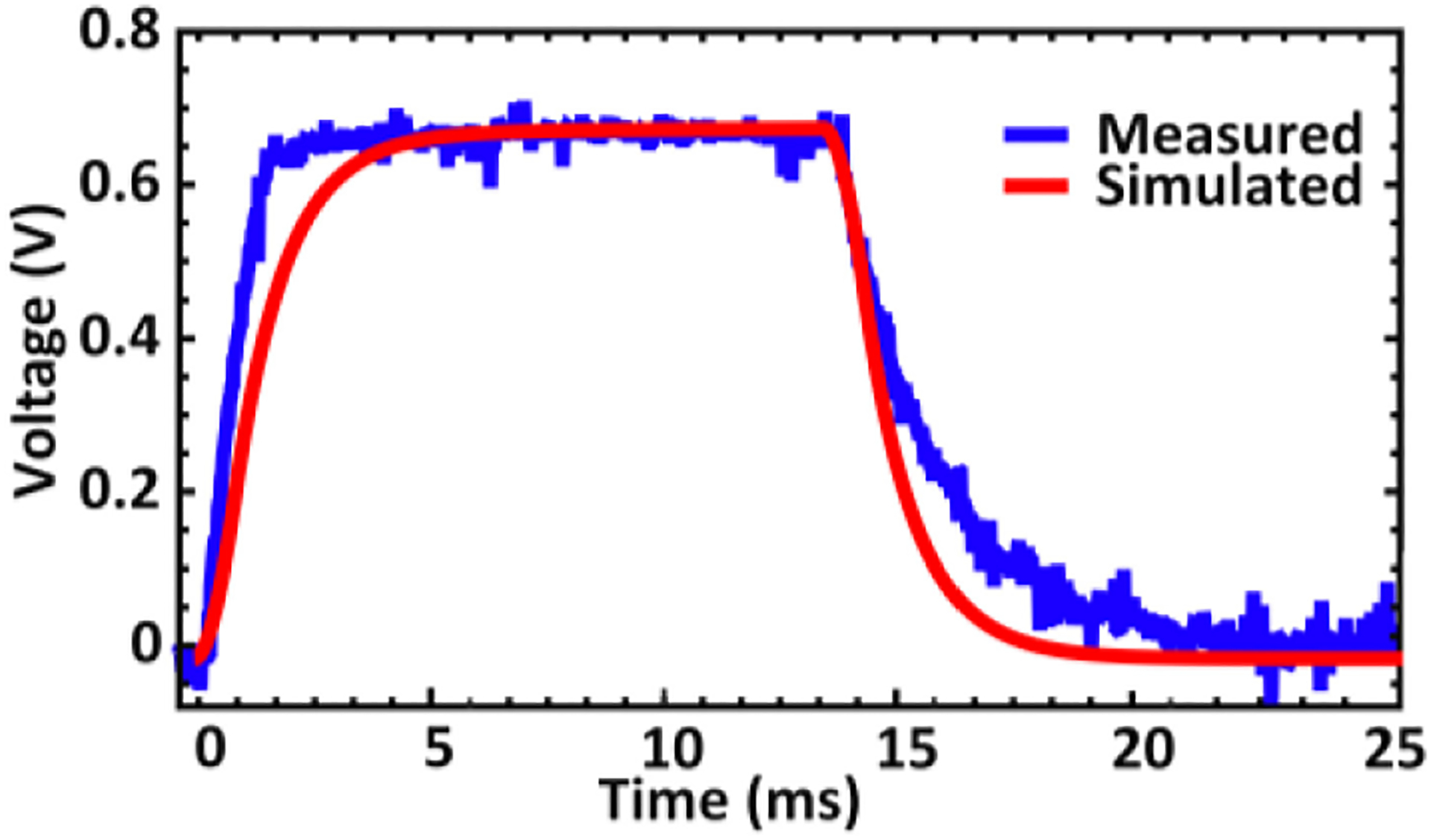
Wireless measurement (Fig. 5) and simulation (Fig. 12) of the energy harvester operation with a pulse-modulated ME antenna excitation and electrode load.

During measurements with the setup in Fig. 5, the energy harvester circuit was able to produce an output rising to 691 mV during the transmission of a pulse-modulated signal with 15 ms pulse width. In order to accomplish wireless power transfer to achieve energy harvesting, the system-level integration of the ME antenna and energy harvester is required. In the presence of coupling losses, the combination of ME antenna with energy harvester chip requires higher input power to achieve the similar performance of standalone wired energy harvester demonstrated in Section IV, in which the measurement was done by directly feeding the power from the signal generator to the input of the energy harvester chip. Similar results as described in Section IV and Fig. 9(b) have been obtained with 2 dB higher input power (to compensate for the extra coupling losses between the ME antenna and the energy harvester within the system) while utilizing the wireless power transfer measurement platform shown in Fig. 5. Fig. 13 displays both the measured and simulated results of the energy harvester operation at the 2.57 GHz resonance mode with electrode load and ME antenna. From the measured output (blue line), a rise time (0 to 95% of the peak voltage) of 2.1 ms and a fall time (100% to 5%) of 4.9 ms can be observed. The red line in Fig. 13 shows the corresponding transient response from the simulation testbench in Fig. 12. It can be seen that the peak voltage reaches 680 mV. The simulated rise and fall times are 3.7 ms and 4.1 ms, respectively. The differences between the measurement and simulation results are in part caused by process variations. Nevertheless, the co-simulation of the energy harvester with the ME antenna model allows to approximately predict the final output during design optimizations.

## VI. MODELING, SIMULATION AND EXPERIMENTAL EVALUATION OF SIMULTANEOUS WIRELESS SENSING AND ENERGY HARVESTING

### A. SINUSOIDAL MAGNETIC EXCITATION

**FIGURE 14.**
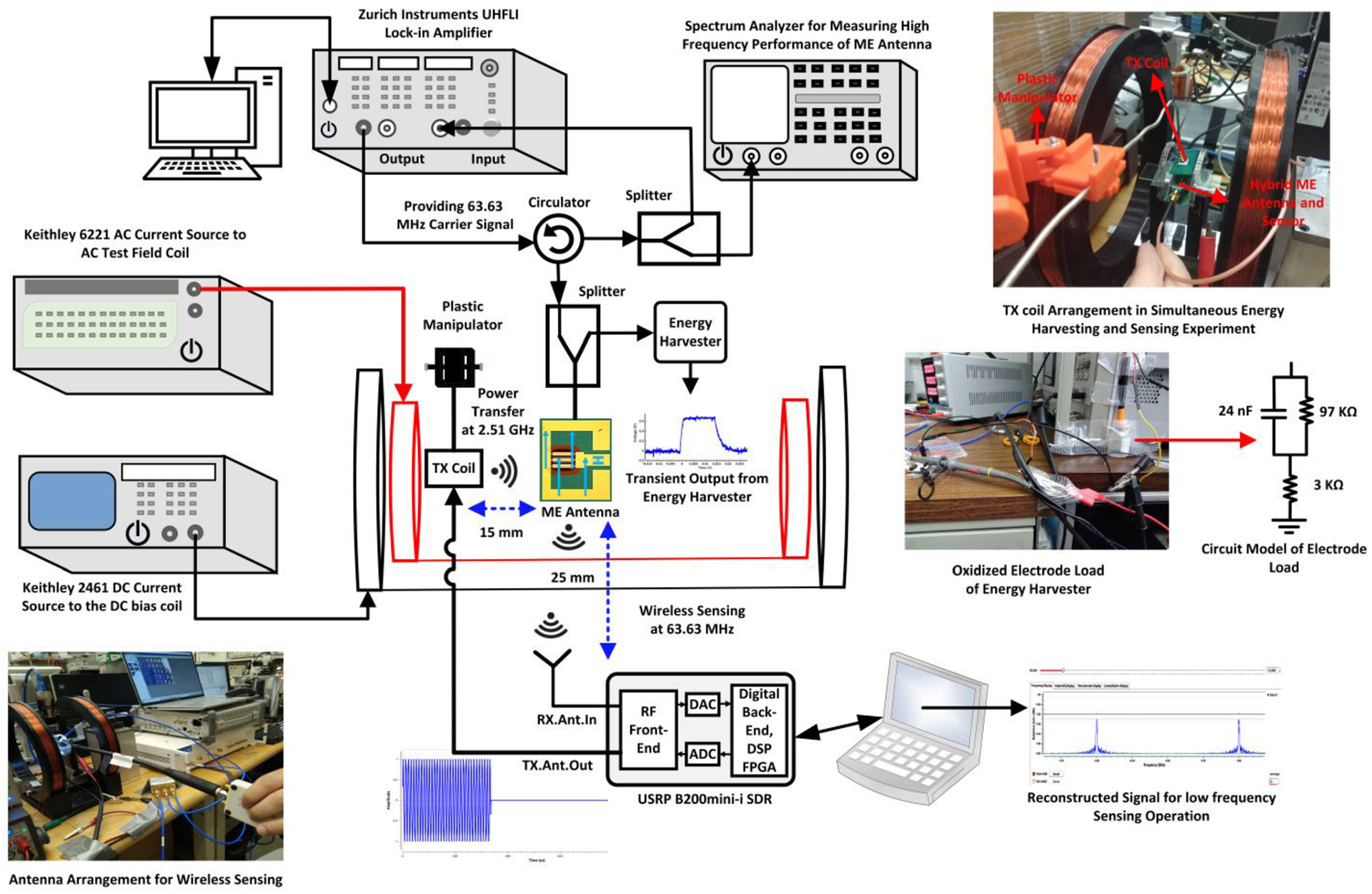
Measurement setup for simultaneous magnetic field sensing and energy harvesting.

Fig. 14 displays the RF measurement setup for prototyping wireless sensing of low-frequency signals with a carrier at 63.63 MHz and concurrent wireless power transfer for energy harvesting at 2.57 GHz. The ME antenna performs the sensing operation while exposed to an external AC magnetic field, which induces a strain in the magnetic heterojunction of the ME antenna. A Keithley 6221 current source is used to generate and provide an AC current with a controllable frequency (for this experiment, the selected frequency is 1 KHz) via a small Helmholtz coil. On the other hand, a DC bias field is applied using a Keithley 2461 DC current source via a larger Helmholtz coil as shown in Fig. 13. The perpendicular orientation of the DC bias field with the resonator increases the magnetoelectric coupling coefficient, resulting an improvement of sensitivity [63]. While the ME antenna is excited by the AC field to be sensed, a lock-in amplifier provides a carrier signal at 63.63 MHz. Due to the nonlinear characteristics of the ME antenna, an electromagnetically amplitude-modulated signal will be produced and transmitted. The circulator and splitter are used to monitor the modulated signal from its returned path by utilizing a SA and Zurich Instruments UHFLI lock-in amplifier to observe the power carried by the modulated signal component as well as the carrier. This gives a clear picture of the power carried by the spectral components and the noise level, which in turn helps to calibrate the SDR for proper signal reception. The front-end gain adjustment technique for optimum power transfer with the SDR and ME antenna is described in [56]. A Radial whip antenna with a frequency range of 30–512 MHz is connected to the receiver port of SDR using a TNC-to-SMA connector to capture the magnetically modulated signal. The received signal is further processed by the GNU Radio software to reconstruct the modulated signal.

In parallel to the magnetic sensing, the simultaneous wireless energy harvesting operation is initiated by providing a pulse amplitude modulated signal of similar power as in Section II-C to the TX coil. A pulse modulated signal was selected to provide electrical pulses for neural stimulation in the future, and the pulse width can be duty cycled according to the required power. The TX coil in Fig. 14 resonates at the same frequency as the ME antenna’s high-frequency resonance mode, which will transmit the signal through a 2.57 GHz wireless link. The distance between the TX coil and ME antenna during simultaneous RF measurement was kept at 15 mm for best wireless power transfer. Upon receiving the RF signal, a strain will be generated in the magnetic material and transferred to the piezoelectric layer of the ME antenna, producing an output power. This generated output power is used for the energy harvesting operation. The energy harvester circuit utilizes an electrode load, as discussed in Section V and displayed in Fig. 12. During the simultaneous sensing and energy harvesting operations, the ME antenna was able to produce a −1 dBm power. There are several losses and interference involved in the measurement, leading to a power level close to −3 dBm at the energy harvester input within the configuration in Fig. 14. We selected a 2.57 GHz frequency for wireless power transfer in proximity of the frequency bands of communication standards around 2.4 GHz, but at a slightly higher frequency to mitigate adjacent interference. The primary degradation is caused by the path loss during both wireless sensing and energy harvesting, as well as the misalignment between the TX coil, the ME antenna and the antenna connected to the receiver port of the SDR. In addition, several components such as the splitter, coupler, adapters, SMA connector, and PCB cause losses during the measurement.

**FIGURE 15.**
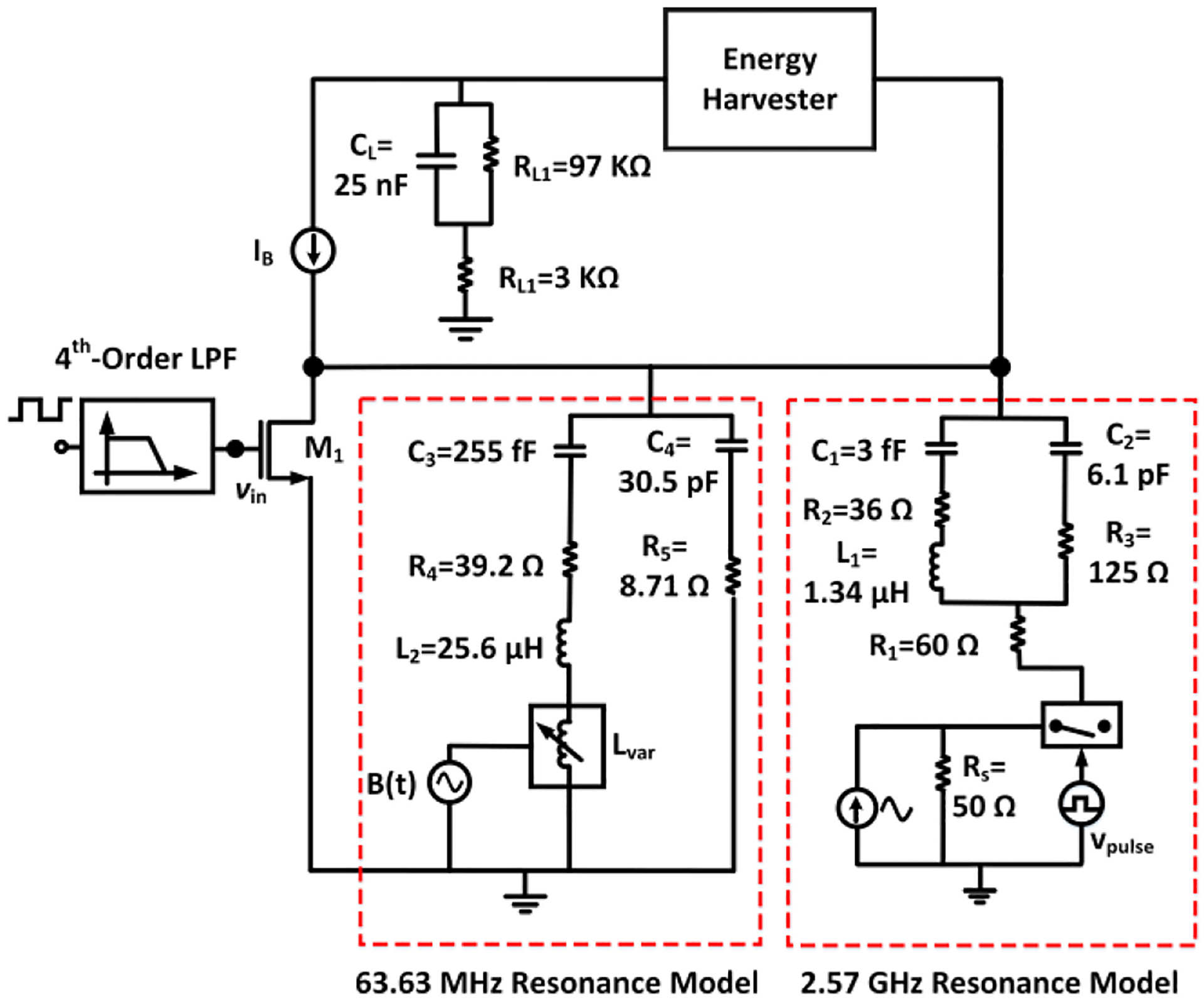
Simulation model for simultaneous sensing and energy harvesting.

**FIGURE 16.**
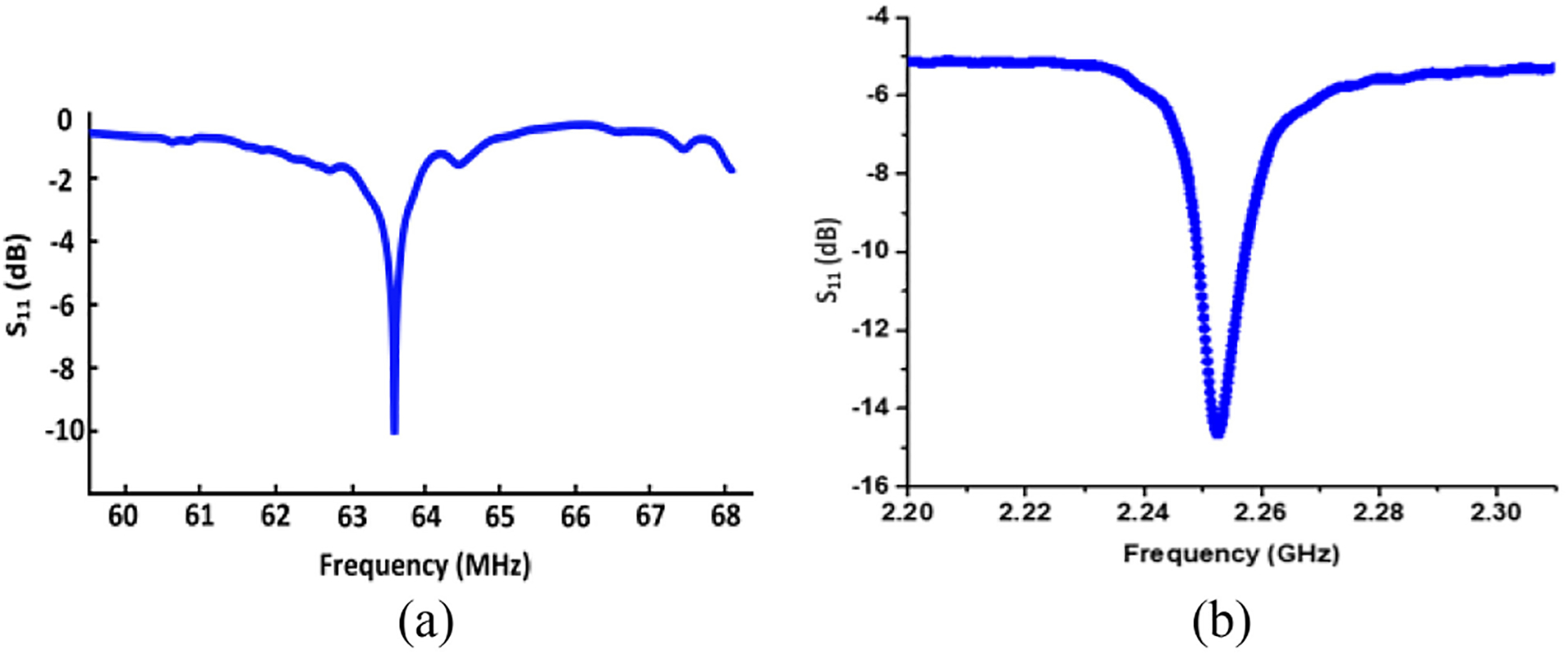
Measured S_11_ parameters of the hybrid ME antenna and sensor: (a) low-frequency resonance mode, (b) high-frequency resonance mode.

**FIGURE 17.**
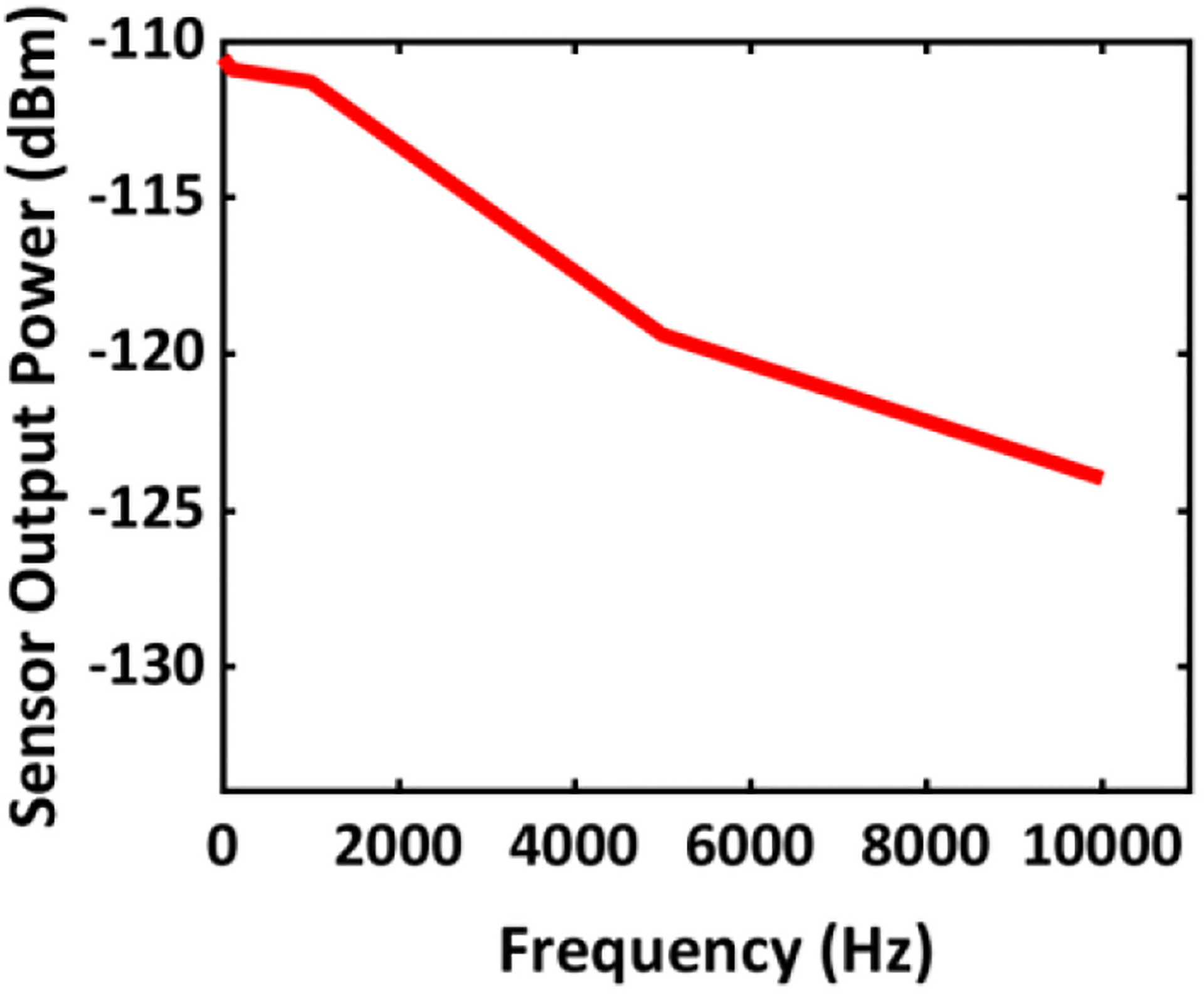
Simulated frequency response of the ME sensor.

Fig. 15 depicts the circuit model for the simultaneous sensing and energy harvesting operations, where the 63.63 MHz ME antenna resonance model and 2.57 GHz ME antenna resonance model represent a single hybrid ME antenna. The circuit models were extracted utilizing the S-parameters during RF measurement at their corresponding resonance frequencies as described in Section III-A. Fig. 16 shows the measured S-parameters of the hybrid ME antenna-sensor during measurement and Fig. 17 displays the simulated frequency response of the ME sensor using the testbench in Fig. 15. To obtain this frequency response, we performed transient analysis to assess the output power carried by the spectral components of the modulated signal at different frequencies. It can be inferred from the plot that the sensor has a relatively flat response for a frequency range from 1 Hz to 1 KHz with less than 1.3 dB variation. As discussed earlier, the ME antenna acts as a sensor while exposed to an AC magnetic field. Similar to the high-frequency mode (described in Section III-A), the MBVD model has been extracted for the low-frequency ME sensing mode. During the experiment, we observed that the effective inductance L_2_ in Fig. 15 is a strong function of the AC magnetic field. Hence, a small change of L_2_ (25.6 *μ*H) in the 63.63 MHz resonance model can emulate small changes of the AC magnetic field. To dynamically model the varying AC magnetic field during simulations, a variable inductor L_var_ (190 nH) has been introduced to the model, which was defined with Verilog-A to realize the time-varying inductance based on the magnetoelectric coupling coefficient of the measured magnetic modulation during the model extraction. This arrangement enables the ideal transient source B(t) in Fig. 15 to emulate the AC magnetic field. For example, the transient signal source B(t) with 0.25 mV amplitude at 1 KHz frequency drives the variable inductor (at the high-impedance control input) to emulate a magnetic field of 0.97 *μ*T, producing an electromagnetically amplitude-modulated signal based on the measured magnetoelectric coupling during the ME sensor characterization. The driver (M_1_) is used to generate a 63.63 MHz carrier signal. An analog filter is driven with a digital input to ease the carrier signal generation in SoC applications. The integration of the 2.57 GHz antenna model captures the energy harvesting operation during simulations. As in measurements, the duty cycle has been kept at 50% during simulations.

Due to the large difference of the 63.63 MHz and 2.57 GHz resonance frequencies of the ME antenna, there are no significant loading effects between the resonance tanks in the circuit-level model. After receiving power from the 2.57 GHz ME antenna model, the energy harvester with electrode load produces same simulated output voltage as shown in Fig. 13, which has been used as the supply voltage for the sensing circuitry. Based on transient simulation, the dynamic power consumption to drive the antenna was found to be 199 *μ*W.

**FIGURE 18.**
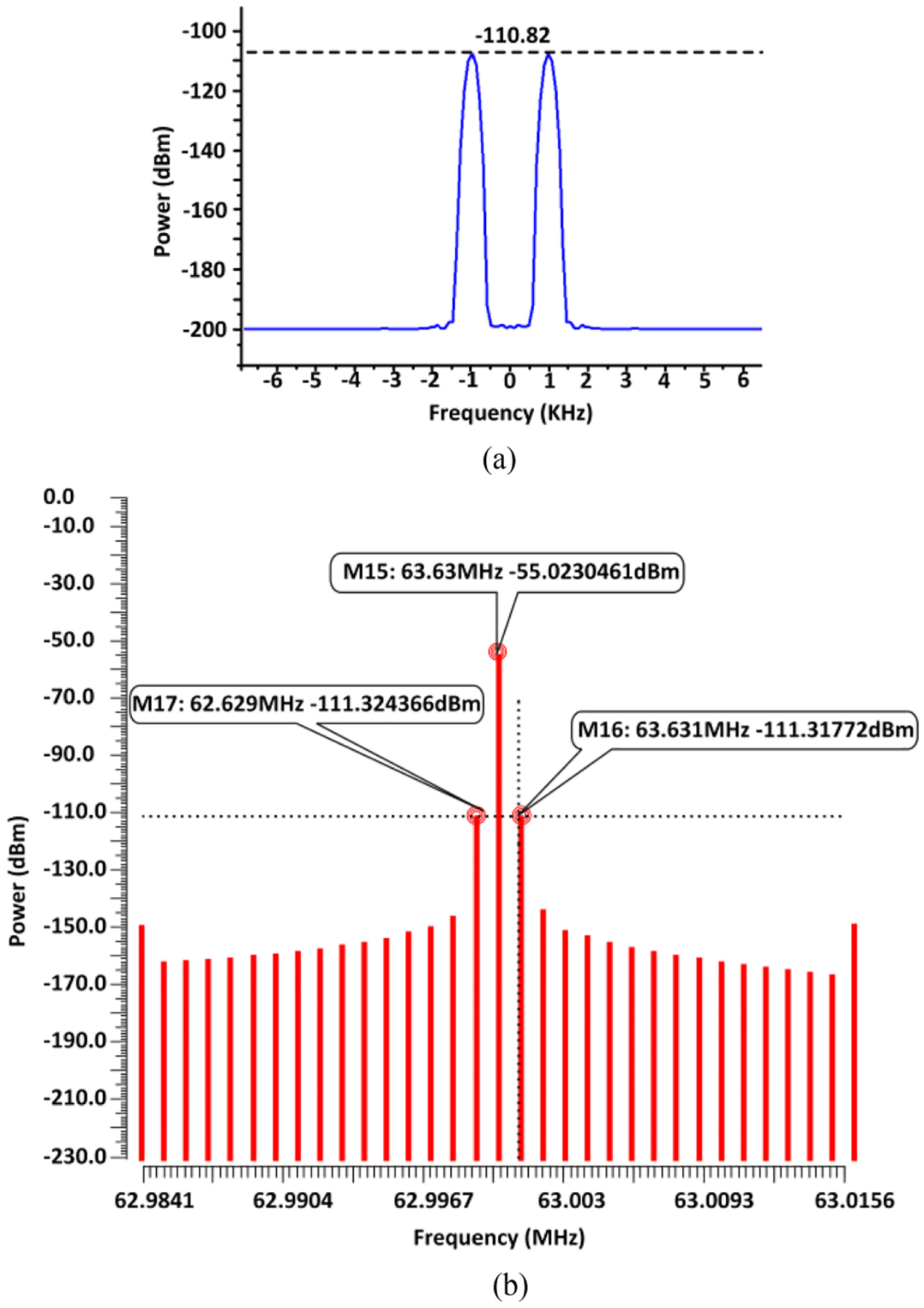
Simultaneous sensing and energy harvesting results: (a) spectral components from RF measurement at 1 KHz offset, (b) sensor output power spectrum from circuit simulation at 1 KHz offset with magnetic field 0.97 *μ*T.

Fig. 18(a) shows a wireless sensing result obtained with the RF measurement platform in Fig. 14, and Fig. 18(b) displays the corresponding simulation result using the testbench in Fig. 15. In Fig. 18(a), the received and reconstructed ultra-low magnitude signal carries −110.82 dBm of power at 1 KHz offset for a 9.4 *μ*T field strength. Fig. 18(b) shows the simulated sensing result, in which the magnetically amplitude modulated signals at 1 KHz offset (from the 63.63 MHz carrier) carries a power of −111.3 dBm. The measurement and transient circuit simulation results are in close agreement with each other.

### B. LIMIT OF DETECTION FOR WIRELESS SENSING

**FIGURE 19.**
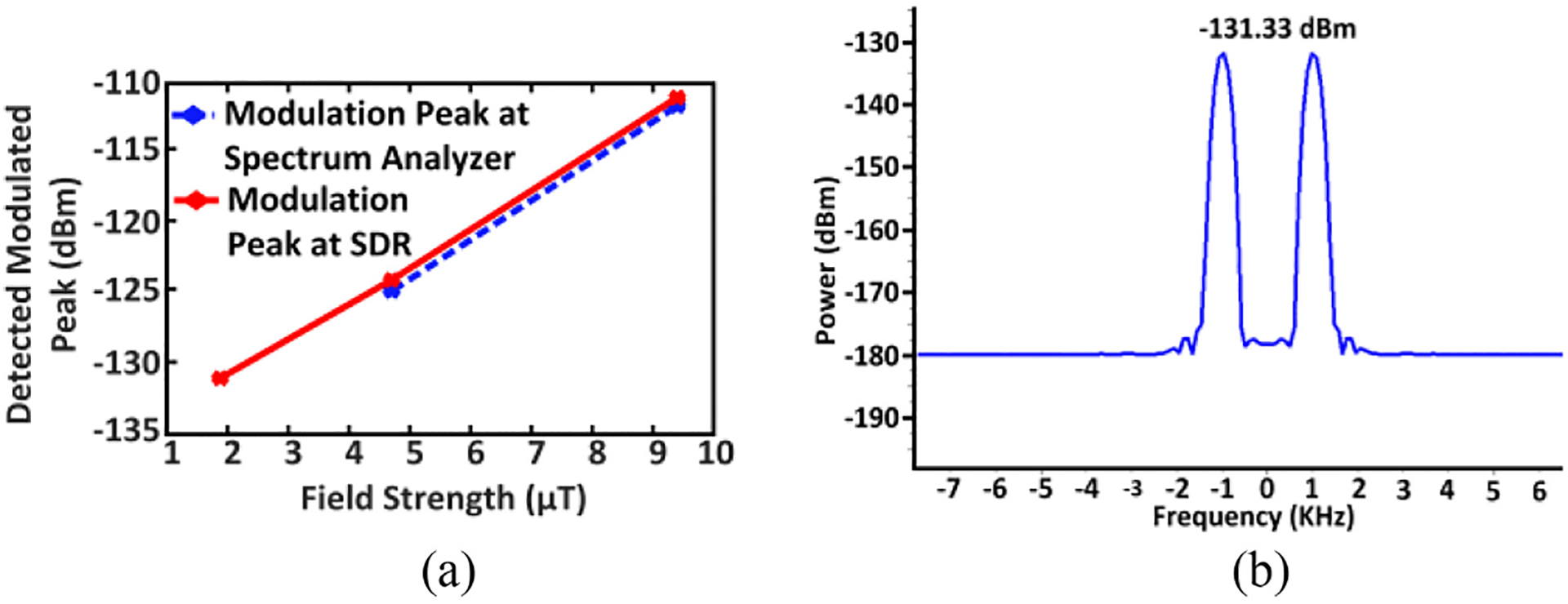
Measured (a) limit of detection (LoD) determination during magnetic field sensing, (b) modulated signal detected by the SDR at 1.88 *μ*T field strength.

To further investigate the performance of the ME antenna as a sensor at 63.63 MHz, the limit of detection (LoD) has been measured utilizing same RF prototyping platform (Fig. 14). During the measurement, the splitter and the energy harvester chip shown in Fig. 14 have been removed to mitigate the losses during wireless transmission. However, the ME antenna is simultaneously exposed to the 2.57 GHz wireless power transfer to validate the hybrid functionality of the ME antenna. The LoD is insightful to estimate the capability of performing sensing within implants based on the described wireless approach. The magnetic field was swept up to 9.4 *μ*T by adjusting the amplitude of the AC current source in the RF test platform. During the measurements, the highest magnetic field produced using the setup in Fig. 15 was 9.4 *μ*T with the maximum AC current from the Keithley 6221 current source at 1 KHz. The spectral power carried by the modulated peaks at 1 KHz offset decreases with the reduction of field strength, while the carrier power provided by the lock-in amplifier was kept constant during the measurements. The reduction of the modulation peaks was simultaneously monitored with the SA and the SDR for comparison. As shown by Fig. 19(a), the detection results with the SA and the SDR are close to each other. However, after a certain point (field strength: 4.7 *μ*T, modulated signal peak: −124 dBm), the SA is unable to detect the signal due to its noise floor limitation; whereas the SDR can still detect the modulated signal peaks with lower field strength down to around 2 *μ*T. Fig. 19(b) shows the SDR-detected modulated signal of −131 dBm at 1 KHz offset for 1.88 *μ*T field strength, which is the LoD with the SDR for this wireless sensing test. The output power carried by the modulated signals below 1.88 *μ*T field strength cannot be detected with the current measurement setup due to the SA and SDR noise floor limitations. The SDR has been used to post-process the wirelessly received signals using the GNU Radio software, where a moving average filter was incorporated to reduce the noise floor in order to detect the amplitude of the spectral components. In addition, the receiver robustness has been improved against the time-varying RF interference by externally calibrating the SDR RF front-end [56].

### C. MAGNETIC EXCITATION WITH ACTION POTENTIALS

Neuronal activity generates small transient currents that produce small NMFs [95], [96]. The ME sensor detects these NMFs signals because its low-frequency mode has high sensitivity to the magnetic field as described in Section II-A. The bias magnetic field modifies the Young’s modulus of f_width_, which leads to an electromechanical resonance frequency change of the ME resonator. In this work, the test setup has been designed to emulate the magnetic field produced by neural activity with the help of the Helmholtz coils shown in Fig. 14. The dimensions and the weight of the USRP B200 mini-I SDR used as external transceiver in Fig. 14 are 83.3 mm × 50.8 mm × 8.4 mm and 24 g, which qualifies as a portable option for an external transceiver during neural stimulation.

**FIGURE 20.**
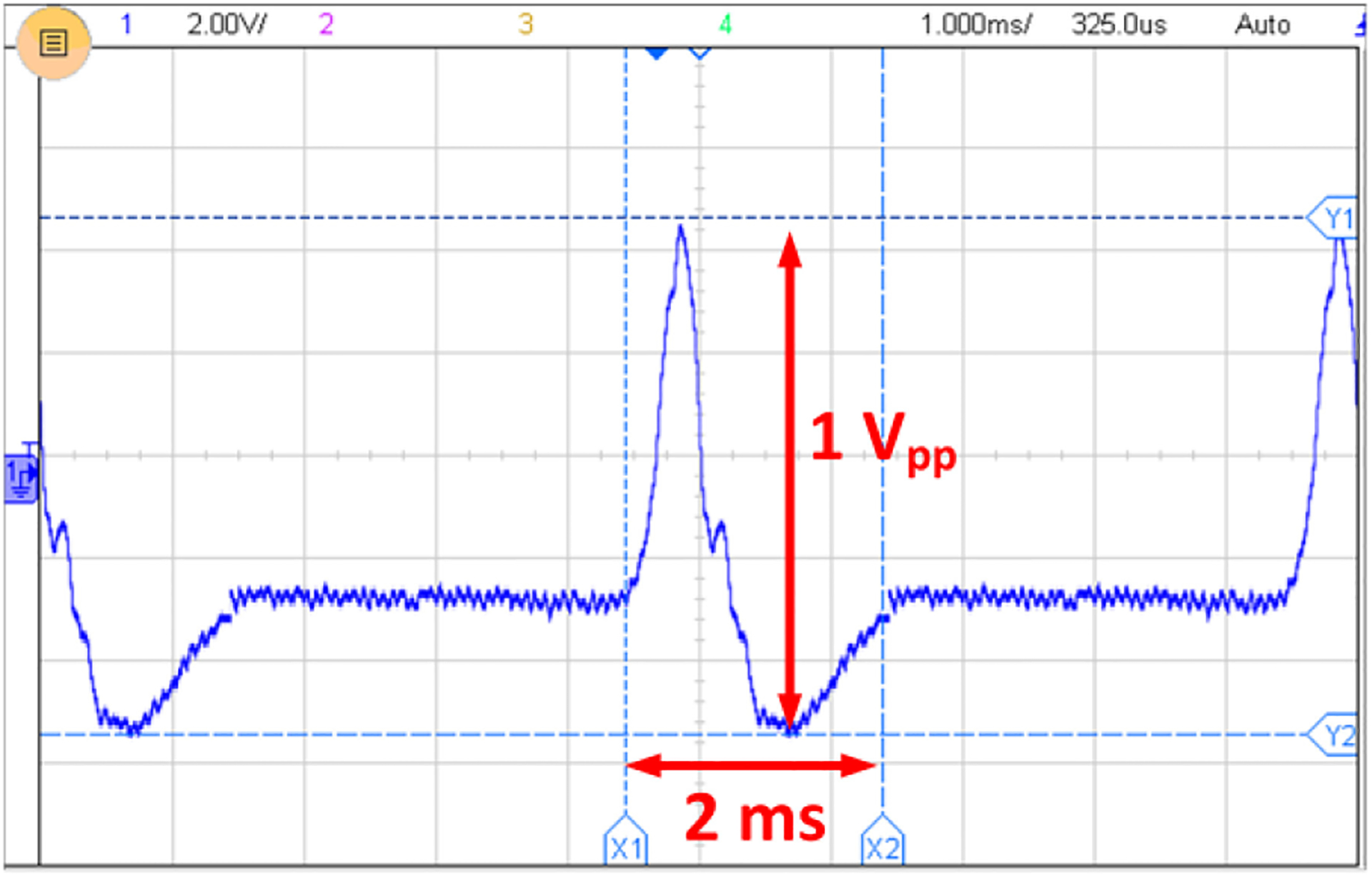
Generated action potential.

**FIGURE 21.**
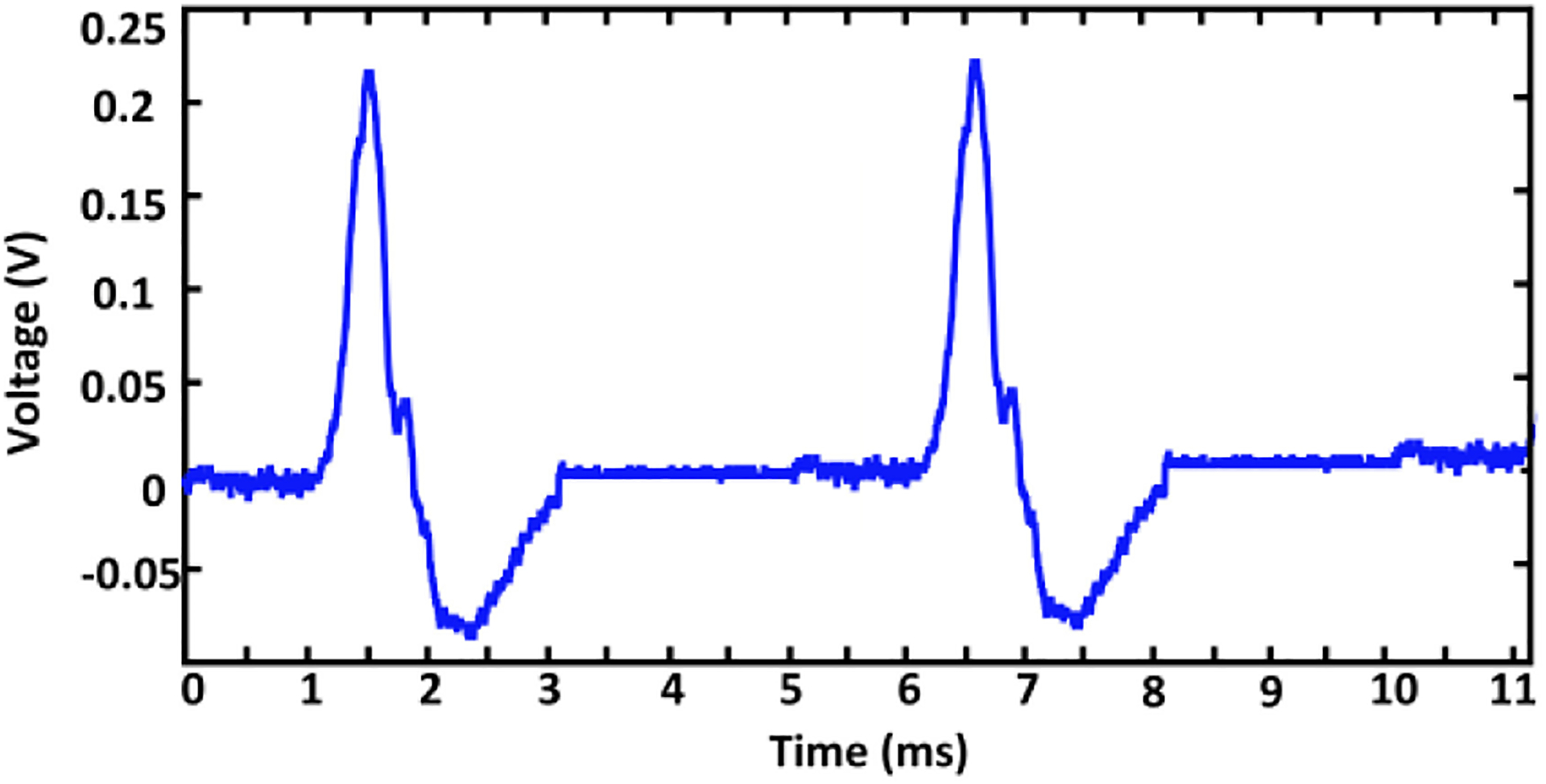
Reconstructed action potentials after digital processing.

To process a realistic test signal, the ME antenna was excited with neural actional potentials to perform magnetic modulation with the low-frequency signals. The test setup utilizes the action potential’s voltage waveform to create the corresponding magnetic field. A 1 V_pp_ signal has a peak magnetic field strength of 4 *μ*T based on the data provided in Fig. 19(a), which emulates the induced magnetic field from the neural activities. The strain of low-frequency action potentials triggers the ME antenna for wireless sensing and transmission over the 63.63 MHz link. The action potential signal shown in Fig. 20 was generated according to references ([31], [35], [97]) using a Siglent SDG 2024X arbitrary waveform generator (AWG). To implement the magnetic modulation with action potential waveforms, the Keithley 6221 AC current source in Fig. 14 was replaced by the AWG, such that the AWG is connected to the small Helmholtz coil to produce modulation through the ME sensing property. The 2.57 GHz link for energy harvesting was simultaneously activated during the sensing of action potentials. As before, the modulated action potentials were transmitted and received by the RF front-end of the SDR. The received signal was further processed through the signal processor of the SDR, leading to the final reconstructed action potential signal displayed in Fig. 21. The reconstructed action potential preserves the pulse span of 2 ms and relative amplitude vs. time characteristics.

## VII. CONCLUSION

A circuit model for the receiver-mode of ME antennas was described, which can be utilized to estimate energy harvesting characteristics during circuit-level simulations. The discussed simulation approach can be used to optimize interface circuits and wireless power transfer efficiency. The proposed integrated model was constructed from extracted ME antenna parameters and auxiliary components to account for excitations with and without pulse modulation. Simulations of the antenna model and loaded energy harvester were compared to measurement results, which revealed the capability to estimate the final output voltage responses with a conventional circuit simulator. After the freestanding simulation and verification of the energy harvesting and sensing, the simultaneous occurrences of both operations were realized with the described wireless platform. Furthermore, both wireless links (63.63 MHz for wireless sensing and 2.57 GHz for wireless power transfer) were modeled for concurrent circuit simulations, which have been verified through measurements. Finally, the ability to perform magnetic modulation and wireless transmission of neural action potential waveforms was demonstrated with the RF prototyping platform.

## Figures and Tables

**TABLE 1.** Wireless power transfer efficiency (PTE) comparison.

Ref.	Type	Distance (mm)	Mode	Frequency (MHz)	Antenna Size (mm^2^)	Efficiency (%)
[66]	IC*	4.6	Single (TX)	1180	0.9	0.002
[67]	CC**	2	Single (TX)	130	400	35
[68]	CC	2	Single (TX)	0.12	800	10
[69]	FF***	200	Single (TX)	2340	9	0.005
[70]	OW****	3	Single (TX)	750	90	16.3
[45]	ME	30	Single (TX)	-	8	0.008
**this work**	ME	15	Dual (TX+RX)	2570	0.25×0.174	0.028

* IC: Integrated Circuit

** CC: Capacitive Coupling

*** FF: Far Field

**** OW: Optical Waves

**TABLE 2.** Measured energy harvester performance parameters.

Parameter	Measurement
S_11_ (dB)	−14.9 dB
Frequency of operation	2.57 GHz
Output voltage at −3 dBm incident power	710 mV
Efficiency	9.5 %
Rise time to steady state (0–95% of peak voltage)	1.9 ms
